# Supplementary material for: Long-read only assembly of Drechmeria coniospora genomes reveals widespread chromosome plasticity and illustrates the limitations of current nanopore methods
Source: Gigascience. 2020 Sep 18;9(9):giaa099. doi: 10.1093/gigascience/giaa099 (PMC7500977; doi:10.1093/gigascience/giaa099)
Supplement: giaa099_GIGA-D-19-00433_Original_Submission [file giaa099_giga-d-19-00433_original_submission.pdf]

GigaScience

Long-read only assembly of Drechmeria coniospora genomes reveals widespread chromosome plasticity and illustrates the limitations of current nanopore methods.

--Manuscript Draft--

**Manuscript Number:**	GIGA-D-19-00433
**Full Title:**	Long-read only assembly of Drechmeria coniospora genomes reveals widespread chromosome plasticity and illustrates the limitations of current nanopore methods.
**Article Type:**	Data Note
**Funding Information:**	
**Abstract:**	Long read sequencing is increasingly being used to determine eukaryotic genomes. We used nanopore technology to generate chromosome-level assemblies for 3 different strains of Drechmeria coniospora , a nematophagous fungus used extensively in the study of innate immunity in Caenorhabditis elegans . One natural geographical isolate demonstrated high stability over decades, whereas a second isolate, not only had a profoundly altered genome structure, but exhibited extensive instability. We conducted an in-depth analysis of sequence errors within the 3 genomes and established that even with state-of-the-art tools, nanopore methods alone are insufficient to generate sequence of a sufficient accuracy to merit inclusion in public databases.
**Corresponding Author:**	Jonathan Ewbank<br><br>FRANCE
**Corresponding Author Secondary Information:**	
**Corresponding Author's Institution:**	
**Corresponding Author's Secondary Institution:**	
**First Author:**	Damien Courtine
**First Author Secondary Information:**	
**Order of Authors:**	Damien Courtine
	Jan Provaznik
	Jerome Reboul
	Guillaume Blanc
	Vladimir Benes
	Jonathan Ewbank
**Order of Authors Secondary Information:**	
**Additional Information:**	
**Question**	**Response**
Are you submitting this manuscript to a special series or article collection?	No
**Experimental design and statistics**<br><br>Full details of the experimental design and statistical methods used should be given in the Methods section, as detailed in our	Yes

Minimum Standards Reporting Checklist. Information essential to interpreting the data presented should be made available in the figure legends.<br><br>Have you included all the information requested in your manuscript?	
**Resources**<br><br>A description of all resources used, including antibodies, cell lines, animals and software tools, with enough information to allow them to be uniquely identified, should be included in the Methods section. Authors are strongly encouraged to cite Research Resource Identifiers (RRIDs) for antibodies, model organisms and tools, where possible.<br><br>Have you included the information requested as detailed in our Minimum Standards Reporting Checklist?	Yes
**Availability of data and materials**<br><br>All datasets and code on which the conclusions of the paper rely must be either included in your submission or deposited in publicly available repositories (where available and ethically appropriate), referencing such data using a unique identifier in the references and in the "Availability of Data and Materials" section of your manuscript.<br><br>Have you have met the above requirement as detailed in our Minimum Standards Reporting Checklist?	Yes

Powered by Editorial Manager® and ProduXion Manager® from Aries Systems Corporation

Manuscript Click here to download Manuscript Courtine.pdf

Long-read only assembly of *Drechmeria coniospora* genomes reveals widespread chromosome plasticity and illustrates the limitations of current nanopore methods.

Damien Courtine[1], Jan Provaznik[2], Jerome Reboul[1*], Guillaume Blanc[3] Vladimir Benes[2] and Jonathan J. Ewbank[1+]

[1]Aix Marseille Univ, CNRS, INSERM, CIML, Turing Centre for Living Systems, Marseille, France

[2]European Molecular Biology Laboratory (EMBL), GeneCore, Heidelberg, Germany.

[3]Aix Marseille Univ., Université de Toulon, CNRS, IRD, MIO UM 110, 13288, Marseille, France

*Current address, Institut de Génetique Moléculaire de Montpellier, Montpellier, France

[+] ewbank@ciml.univ-mrs.fr

Abstract

Long read sequencing is increasingly being used to determine eukaryotic genomes. We used nanopore technology to generate chromosome-level assemblies for 3 different strains of *Drechmeria coniospora*, a nematophagous fungus used extensively in the study of innate immunity in *Caenorhabditis elegans*. One natural geographical isolate demonstrated high stability over decades, whereas a second isolate, not only had a profoundly altered genome structure, but exhibited extensive instability. We conducted an in-depth analysis of sequence errors within the 3 genomes and

established that even with state-of-the-art tools, nanopore methods alone are insufficient to generate sequence of a sufficient accuracy to merit inclusion in public databases.

Background

Drechmeria coniospora is an obligate parasitic fungus belonging to the order of Hypocreales. This fungus forms spores that adhere to the cuticle of a range of different nematodes to infect them [1]. We adopted *D. coniospora* strain ATCC-96283, derived from a strain isolated in Sweden, as a model pathogen for *Caenorhabditis elegans* 20 years ago [2]. We have cultured this strain, referred to here as Swe1, continuously since then, using it to understand innate immune mechanisms in its nematode host [3,4].

As part of our characterization of the interaction between *D. coniospora* and *C. elegans*, in 2013, we extracted DNA from our laboratory strain of the time (referred to here as Swe2), and determined its genome. Despite attempts to complete the assembly, the Swe2 genome remained fragmented, with an N50 of 3.86 Mb [5]. In addition to the genome of Swe2, a second *D. coniospora* genome is available (referred to here as Dan2) [6], derived from a strain related to a Danish isolate (Dan1; Figure 1). Although corresponding to a chromosome level assembly, this latter genome still contains large inserts (up to 500 kb) of undetermined sequence. In this study, we used Oxford Nanopore Technology (ONT) long-read sequencing to assemble complete fungal genomes. This revealed that the 2 isolates (Swe1 and Dan1) display strikingly different levels of genomic stability. It also allowed us to characterize the continuing challenges to using only ONT long-read sequencing for genome assembly.

Result

An all-against-all *in silico* genome comparison of the 2 publicly available *D. coniospora* genome sequences, for Dan2 [6] and Swe2 [5], indicated the presence of extensive genomic rearrangements (Figure 2A). These could reflect real differences or assembly errors in one or both genomes. We directly confirmed one major rearrangement by PCR (Figure 2B, C), suggesting that the differences could be real. To characterise this genomic plasticity, we determined the genomes of 3 strains related to the 2 that had been sequenced previously (Figure 1). We used ONT nanopore sequencing to generate long reads and current assembly tools to construct chromosome level assemblies for all 3 strains (Supplementary Fig. S1). Manual curating allowed complete mitochondrial genomes to be predicted from the concatenated assembly generated by Canu [7].

All 3 nuclear genomes were divided in 3 similarly sized chromosomes, an unusual arrangement for such a fungus, as previously noted by Zhang *et al*. for Dan2 [6] . For the 2 strains related to Swe2, there was almost complete synteny of their nuclear genomes. Inspection of the one anomalous region in Swe1 where synteny broke down revealed that it was supported by only one long read (215 kb) and corresponded to a local discontinuity in the read coverage, as well as a break in the alignments between Canu-generated contigs and unitigs. All these factors indicated that this was an assembly artefact with a contig misassembled erroneously on the basis of an individual very long chimeric read (Supplementary Fig. S2). The same was true for the distinct unique non-syntenic region of the Swe3 assembly (Supplementary Fig. S3). Notably the original chimeric read on which this misassembly was based was not detected by

current tools, although in reads recalled by Guppy (see Methods) it was flagged as anomalous. This is an indication of the continuing improvement to base-calling tools. When these regions were manually corrected, Swe1 and Swe3 were entirely collinear (Figure 3A).

These 2 genomes have 3 almost equally sized chromosomes (8.5 Mb, 11.6 Mb, 11.6 Mb), each with identifiable telomeric [8] and centromeric regions, indicating that the overall genome structure has remained constant over 20 years of laboratory culture. This allowed us then to use the Swe1 sequence to scaffold the fragmented Swe2 genome (Figure 3B). To our great satisfaction, we were able to produce an entirely collinear chromosome scale assembly. Thus it appears that there were no assembly errors in the published Swe2 genome, it was simply incompletely scaffolded. This applies equally to the genomic regions containing copies of some mitochondrial genes that we previous suggested might indicate assembly errors. They were revealed to be accurate; *D. coniospora* has nuclear paralogous copies of 10 mitochondrial protein-coding and 15 tRNA genes. These results give further support to the existence of long-term stability of the genome of the Swe1 isolate.

A whole genome comparison between Swe1 and Dan2, however, revealed multiple and extensive genome rearrangements, involving intra- and inter-chromosomal translocations and inversions (Figure 3C). When we compared Dan1 and Dan2, we were surprised to find 2 major events of reciprocal exchange of chromosome ends, and an intra-chromosomal inversion (Figure 3D). These events were supported in a coherent and consistent manner by all the available data (Supplementary Fig. S4). As the Dan2 assembly is of high confidence, supported by long reads and optical mapping

[6], given the short time of *in vitro* culture that separates it from Dan1, this suggests that the genome of the Dan1 isolate is not stable.

In alignments of the sequence of Swe1, generated using only nanopore reads, with that of Swe2, there were stretches of complete nucleotide identity extending over more than 25 kb. This is a testament to the general reliability of nanopore sequencing. We wished, however, to determine whether the 3 new genomes were of sufficient quality to allow accurate gene prediction. We therefore identified the complete set of proteins identical in Swe2 and Dan2 corresponding to single copy, single exon genes (see Methods). These would be expected to be present in the newly assembled Swe1, Swe3 and Dan1 genomes. Indeed, using these 305 genes as a query, we could identify homologous sequences for each in all 3 genomes. Only around 2/3 of the corresponding genes, however, were predicted to encode full-length proteins in the Swe1 and Swe3 genomes, falling to less than half for Dan1 (Figure 4A). While nanopore reads are very useful for genome assembly, they suffer from a high error rate, especially in homopolymer stretches. Sequence quality can be improved using polishing tools that aim to ameliorate consensus sequences generally by going back to raw reads and applying integrative algorithms [9] . In our case, applying current best practices, while providing a moderate improvement (up to 25% in the best case), did not take the prediction level beyond 85% accuracy. Again, the quality of the prediction seen with the Dan1 genome was strikingly lower than the other 2 genomes (Figure 4A).

Inspection suggested that the majority of errors were in homopolymer sequences, as expected, with nucleotide insertions and deletions leading to alterations of the reading frame. To investigate this poor homopolymer predictive performance systematically,

we computed the number of G/C or A/T homopolymer stretches of at least 4 nucleotides for each of the 305 genes. We plotted these values, indicating the proportion of genes that encoded the expected full-length predicted protein for each of the 3 genomes. While there was the expected inverse relationship between accuracy and the number of homopolymer stretches, there were striking exceptions. Curiously some of these exceptions were specific to a single genome (Figure 4B-D). Further, and unexpectedly, polishing introduced more nucleotide insertion errors than deletions, frequently on the basis of tenuous read support. Overall, however, there was no obvious pattern to explain why errors were introduced, given the underlying reads used to build the consensus sequence (Supplementary Table S1).

During the inspection of the assembled and polished genomes, we found two other types of anomalies. The first concerned the regions flanking the nuclear genomic copies of mitochondrial genes, where polishing added short extraneous low complexity sequences (average length 15 nt, mainly As or Ts), for which, surprisingly there was no sequence support from the reads used by the assembler (Figure 5A). In the second case, for the Swe3 genome, a large (ca. 10 kb) region, with a complex sequence, well supported by available reads and present in the Canu-generated contigs and unitigs, was inexplicably excluded from the initial Canu assembly and only imprecisely restored by polishing (Figure 5B). These few regions were identified because of discontinuities in the depth of read coverage, which otherwise was remarkable constant across the complete genomes. With the resolution of these assembly errors, if not of sufficient sequence quality to allow accurate gene prediction, we were able to generate genomes of high overall structural quality using ONT long reads only.

Discussion and conclusion

Previous genome assemblies for *D. coniospora* required a combination of sequencing approaches [5,6]. Here, using only long reads and Canu, we were able to generate chromosome-scale assemblies. The rare misassembled contigs, formed because of single very long chimeric reads, could be detected by read coverage anomalies and comparisons with unitigs, suggesting that solutions to avoid their creation could be implemented within Canu.

One clear and well-established advantage of using long reads is the possibility of resolving very extended stretches of complex tandem repeats (VeCTRs) [10] and other repetitive sequences including centromeres. These correspond to most of the breaks in the continuity of the published Swe2 genome. In addition to acrocentric regional centromeres, Zhang *et al*. reported the presence of a vestigial centromere from a putative chromosomal fusion event [6]. These were also found in the fully assembled Swe1 and Swe3 genomes, indicating that chromosomal fusions were present in the common ancestor of the Swe1 and Dan1 strains.

For Swe1, Swe3 and Dan1 we were able to reconstruct complete mitochondrial genomes, with features typical of fungi of the order Hypocreales. On the other hand, unlike Dan1 (and Dan2), the nuclear genomes of Swe1 and its derivatives Swe2 and Swe3, contained different numbers of copies of sequence very similar to parts of their own mtDNA. This type of event, more readily detectable with long reads, has been described in other fungal genomes [11] and must have occurred after the divergence of Dan1 and Swe1. Despite this genome plasticity, even after 20 years of continuous laboratory culture the Swe1 and Swe3 genomes were entirely collinear. This contrasts with the rearrangements seen between the Dan1 and Dan2 genomes that in principal

should be from strains that have had little opportunity to diverge (L. Castrillo, Curator, ARS Collection of Entomopathogenic Fungal Cultures, personal communication). It will be interesting in the future to characterize the borders of the sites of reorganization to try to identify common sequence elements that could be involved, as well as the reasons for the marked difference in genomic stability between Dan1 and Swe1.

The accuracy of ONT long read sequencing is increasing because of improvements in the chemistry used, signal detection as well as base-calling [12]. Indeed, research groups are publishing and submitting to public sequence databases genomes for fungi, plants and animals based on nanopore sequencing alone (52 Eukaryotes and 99 Bacteria in GeneBank release 234 from the 10/14/2019). Despite good read depth, however, our assemblies were not of sufficient quality at the nucleotide level to allow accurate gene prediction. We therefore have chosen to make our sequence data accessible via our institutional website rather than pollute a public database like GenBank with an inaccurate nucleotide sequence, since low quality genomic sequences compromise the accuracy of sequence similarity searches in public databases. We expect to release the genomes to GenBank only after future short-read polishing.

Regarding the apparent sequence errors, the overwhelming majority were in homopolymer sequences. As noted above, these errors were not consistent across the sequenced genomes; even between Swe1 and Swe3 there were instances of widely differing rates of errors in orthologous genes, despite very similar underlying reads. Indeed there was no clear pattern in the inaccuracies, which will render bioinformatics approaches to remedy this problem more difficult. On the other hand, the errors were more often over-prediction of homopolymer length, despite having a majority of reads

supporting the correct sequence. It is possible that polishing tools have not kept pace with improvements in base-calling, leading to an over-compensation in the inference of homopolymer length.

In conclusion, long read sequencing provides a powerful way to assemble fully complex genomes with limited manual curation. In our case, it has revealed new information about genome plasticity in *D. coniospora* and provided a backbone that will permit future detailed study to characterize gene evolution in this important model fungal pathogen.

Methods

DNA extraction:

D. coniospora spores were cultured in liquid NGMY medium [13] at 37°C for 5 days. Fungal DNA was extracted according to a published protocol [14], with the following modifications: instead of centrifugation to collect DNA after precipitation with isopropanol, we recovered the DNA filaments with a glass hook, washed and dried them as described [15] and resuspended the DNA without agitation in TE.

Nanopore sequencing library preparation:

Libraries were prepared for sequencing on GridION with the ligation sequencing kit SQK-LSK109. The GridION sequencing was run on flowcell version FLO-MIN 106.

Basecalling, adaptor trimming and chimeric read detection:

For a first assembly, reads were basecalled at the EMBL using Guppy v1.5.1 (Oxford Nanopore Technologies). For subsequent polishing, we used Guppy v3.0.3 (with parameters -c dna_r9.4.1_450bps_hac.cfg), then adaptor were then trimmed with Porechop v0.2.4 [16] with default parameters. YACRD v0.5.1 [17] with the subcommand chimeric and the option --filter detected was used to remove chimeric reads.

Whole genome alignments:

Genomes were aligned using LAST v979 [18]. A database was first generated (last-db -cR01), and then lastal and last-dotplot with default parameters were used to generate

respectively an alignment file and a dot-plot. For the circular visualization of genome alignments, we used the command lastal with -f BlastTab parameter, and parsed the alignment with a custom python script (available on request) to generate the links file needed by Circos [19].

Read mapping:

Validation of genomes during and after assembly involved rounds of read mapping. Reads were aligned with Minimap2 v2.16r922 [20] (with parameters -ax map-ont). The resulting mapping file was processed with Samtools v1.9 [21] to obtain a sorted BAM file (samtools view -bS -q 1 -F 4; samtools sort; samtools index). Mapping results were visualized with IGV v2.5.0 [22].

Genome assembly:

Assemblies were performed with Canu v1.7 [23] and the parameters useGrid=False, genomeSize=30m, correctedErrorRate=0.16 with reads basecalled by Guppy v1.5.1. For the manual curation of the assemblies, we generated whole assembly alignments and dot-plots of Swe1, Swe2 and Swe3 two by two. For Swe1 and Swe3, Canu contigs were ordered by synthesizing the results from the 3 possible all-against-all alignments. To confirm a link between two contigs, we employed the following strategy: when a contig of the Swe1 assembly spanned two contigs of Swe3, long reads of Swe1 present in this spanning area were extracted from the Swe1 corrected and trimmed reads provided by Canu. Then this set of reads was mapped on Swe2 and Swe3 assemblies. The two targeted contigs of Swe3 were considered 'linked' if different parts of several unique reads mapped on the two Swe3 contigs ends. If the reads that supported the

link had different mapping orientation (forward or reverse), one contig was complemented before the last step (see *Solve links between contigs*) to ensure a correct orientation of the final chromosome.

To guide correct assembly, we also searched for centromeres in the contigs. They were identified as highly duplicated regions in the all-against-all alignment dot-plots produced by LAST. The identification of the repeated canonical telomeric sequence $(TTAGGG)_n$ [8] and its reverse complement $(CCCTAA)_n$ at the beginning or end of certain contigs allowed the identification of chromosome ends. The Dan1 assembly was manually curated using a similar strategy with the Dan2 genome as a reference.

Solving links between contigs:

Overlaps between linked contigs were identified by a BLASTn [24] alignment of their last 100 kb. Any duplicate sequence was trimmed out from one contig and both contigs were joined. The inferred junction was then validated by verification of the underlying read support. For the linked contigs that did not overlap, the sequence in the gap was extrapolated from the reads that matched and extended the ends of contigs, on the basis of alignments at the last 1 kb of each contig. These sequences were aligned with MAFFT v7.427 [25]. The alignment was visualized with SeaView [26], and only the portion of the alignment strictly between the two contigs sequences was kept. Seaview also generated a consensus sequence (on the basis of 60 % sequence identify by default). The resulting sequence was inserted between the two contigs to link them and the supposed continuity verified by a further cycle of read mapping.

Assembly polishing:

Genome polishing was carried out with 2 or 4 iterative runs of RaCon v1.4.2 [27] and parameters -m 8 -x -6 -g -8 -w 500, by a run of Medaka v0.8.1 (Oxford Nanopore Technologies) with the parameter -m r941_min_high.

Mitochondrial genome circularization:

Canu assembles small circular elements as contigs with tandem duplications of the element. We resolved the mitochondrial genomes as recommended by Canu's authors [7]. MUMmer suite v4.0.0.beta2 [28] was used to align the contig identified as the putative mitochondria on itself with NucMer and parameters --maxmatch --nosimplify . Coordinates of a full copy were identified with the show-coords command and -lrcT parameters.

PCR:

PCR was carried out to test a genome rearrangement between Swe2 and Dan2 genomes, with primers P1F (GAGATATCGAACGTCGCATGG), P1R (ACATCAAGCCTTTGTCGAGGA), and P3F (GCTCAGGACCGACGTACAAG). PCR reactions were run according to the GoTaq® G2 Flexi DNA polymerase instructions (Promega), with 50 ng of template DNA, 1 mM of each forward and reverse primers, in a final volume of 25 μL. The reaction started by initial denaturation at 95°C for 2 min, followed by 30 amplification cycles (95°C for 30 sec, 60°C for 30 sec and 72°C for 30 sec), and a final elongation for 5 min at 72°C.

Defining a set of 305 identical proteins:

Identical proteins shared by the two *D.* coniospora genomes available (Swe2 and Dan2) were recovered using a reciprocal best BLAST [24] hit strategy on the two proteomes. Proteins that were duplicated in one or both genomes were filtered out. The set was further refined by only retaining proteins corresponding to mono-exonic genes.

Assessment of gene sequence in ONT-only assemblies:

tBLASTn searches were run using the amino-acid sequence of the set of 305 identical proteins against the different nanopore only assemblies. A gene was considered as correct if the query coverage, *i.e* the ratio of alignment length over the query length, was equal to 1.

Data availability

Polished genomes of the strains Swe1, Swe3 and Dan1 are available on our institute website (http://www.ciml.univ-mrs.fr/applications/DC/Genome.htm).

Funding

Supported by institutional grants from the Institut national de la santé et de la recherche médicale, Centre National de la Recherche Scientifique and Aix-Marseille University to the CIML, and the Agence Nationale de la Recherche program grant (ANR-16-CE15-0001-01), and "Investissements d'Avenir" ANR-11-LABX-0054 (Labex INFORM), ANR-16-CONV-0001 and ANR-11-IDEX-0001-02, and funding from the Excellence Initiative of Aix-Marseille University - A*MIDEX.

Acknowledgments

The authors thank Yuquan Xu and Liwen Zhang for providing access to the raw sequencing data for Dan2, Xing Zhang for help in preparing DNA samples, Lionel Spinelli for informatic support and Nathalie Pujol and Laurent Tichit for comments.

Supplementary data

supplementary_figures.pdf contains 4 supplementary figures.

supplementary_table_1.csv is a table recording read support, in Swe1, Swe3 and Dan1 assemblies, for the predicted correct sequence for each homopolymer stretch in the genes corresponding to 10 protein of the 305 candidate identical proteins.

supplementary_methods.pdf contains additional methodological details.

Figure legends

Figure 1. An overview of *D. coniospora* strain isolation and culture history. A strain of *D. coniospora* collected from Denmark in 1982 at the latest was deposited at the CBS-KNAW culture collection, now held by the Westerdijk Fungal Biodiversity Institute as CBS615.82. It was transferred in 1987 to the ARS Collection of Entomopathogenic Fungal Cultures (as ARSEF 2468) and then re-isolated in 2001 as ARSEF 6962. A second strain collected from Sweden was deposited at the American Type Culture Collection as ATCC 96282. It has been cultured through serial passage in *C. elegans* continuously since 1999.

Figure 2. Inter-chromosomal rearrangements between strains Swe2 and Dan2.

A. Circos plot representing regions >6 kb that are very similar between Dan2 (left – olive) and Swe2 (right – light blue) assemblies as determined by an all-against-all LAST analysis. Swe2 contig numbers are the last two digits of the accession ID (shown in B), preceding the suffix. Red and dark blue rectangles represent rearrangement junctions probed by PCR. B. Conceptual design of the PCR primers. C. Amplicons from the PCR were visualized after electrophoresis. Each pair gave one specific band of the expected size. The color code is the same for the 3 panels.

Figure 3. Synteny among the genomes of 5 *D. coniospora* strains.

Circos plot representing regions >20 kb that are very similar between assemblies as determined by all-against-all LAST analyses. Each assembly is shown at the same scale and in the same order and orientation across panels.

Figure 4. Evaluation of sequence errors in the 3 new genomes.

A. Number of correct genes (based on length of the corresponding predicted protein) among 305 conserved genes, for the 3 new genomes, in the initial assembly and after

two different polishing strategies. B, C, D. Scatter plots of homopolymer composition (A/T or C/G) and accuracy among the same 305 conserved genes for Dan1 (B), Swe1 (C) and Swe3 (D). The dot size is proportional to the number of genes, and the colour indicates the proportion of genes predicted to be correct. Red and purple arrows highlight two particular cases, among many, where homopolymer errors are only present in one genome.

Figure 5. Sequence anomalies introduced by assembly and/or polishing tools.

A. A comparison of one small region of the Swe3 sequence before (top) and after polishing (RaCon x4 and Madaka; bottom). As indicated by the orange line, long stretches of A and T homopolymers are introduced by polishing, in the absence of coherent read support. B. From top to bottom, the assembly produced by Canu excludes a region of around 10 kb, despite strong read support. After 2 and 4 iterations, RaCon progressively filled the gap. Medaka then introduced an insert of roughly the correct size, but of aberrant sequence composition as depicted by the pie chart.

For each panel, the height of the boxes in the top line indicates the read coverage for each base. A grey box indicates full agreement with the consensus sequence, otherwise the colour indicates the proportion of read support for each nucleotide (G, tan; C, blue; A, green; T, red). Below this, the ONT reads that align in forward (pink) and reverse (blue) orientation are shown as lines. A coloured letter or purple rectangle show a difference (nucleotide variant or insertion in reads, respectively) in the read's sequence compared to the genome sequence.

References

1. Jansson HB, Jeyaprakash A, Zuckerman BM. Differential Adhesion and Infection of Nematodes by the Endoparasitic Fungus *Meria coniospora* (Deuteromycetes). Appl Environ Microbiol. 1985;49:552–5.

2. Pujol N, Link EM, Liu LX, Kurz CL, Alloing G, Tan MW, et al. A reverse genetic analysis of components of the Toll signalling pathway in *Caenorhabditis elegans*. Curr Biol. 2001;11:809–21.

3. Dierking K, Polanowska J, Omi S, Engelmann I, Gut M, Lembo F, et al. Unusual regulation of a STAT protein by an SLC6 family transporter in *C. elegans* epidermal innate immunity. Cell Host Microbe. 2011;9:425–35.

4. Labed SA, Omi S, Gut M, Ewbank JJ, Pujol N. The pseudokinase NIPI-4 is a novel regulator of antimicrobial peptide gene expression. PloS One. 2012;7:e33887.

5. Lebrigand K, He LD, Thakur N, Arguel M-J, Polanowska J, Henrissat B, et al. Comparative Genomic Analysis of *Drechmeria coniospora* Reveals Core and Specific Genetic Requirements for Fungal Endoparasitism of Nematodes. PLOS Genet. 2016;12:e1006017.

6. Zhang L, Zhou Z, Guo Q, Fokkens L, Miskei M, Pócsi I, et al. Insights into Adaptations to a Near-Obligate Nematode Endoparasitic Lifestyle from the Finished Genome of *Drechmeria coniospora*. Sci Rep. 2016;6:23122.

7. Canu FAQ — canu 1.8 documentation [Internet]. [cited 2019 Nov 15]. Available from: https://canu.readthedocs.io/en/latest/faq.html#my-circular-element-is-duplicated-has-overlap

8. Schechtman MG. Characterization of telomere DNA from *Neurospora crassa*. Gene. 1990;88:159–65.

9. Senol Cali D, Kim JS, Ghose S, Alkan C, Mutlu O. Nanopore sequencing technology and tools for genome assembly: computational analysis of the current state, bottlenecks and future directions. Brief Bioinform. 2018;20:1542–59.

10. Eccles D, Chandler J, Camberis M, Henrissat B, Koren S, Le Gros G, et al. De novo assembly of the complex genome of *Nippostrongylus brasiliensis* using MinION long reads. BMC Biol. 2018;16:6.

11. Hazkani-Covo E, Zeller RM, Martin W. Molecular Poltergeists: Mitochondrial DNA Copies (numts) in Sequenced Nuclear Genomes. PLoS Genet. 2010;6:e1000834.

12. Wick RR, Judd LM, Holt KE. Performance of neural network basecalling tools for Oxford Nanopore sequencing. Genome Biol. 2019;20:129.

13. He LD, Ewbank JJ. Polyethylene Glycol-mediated Transformation of *Drechmeria coniospora*. Bio-Protoc. 2017;7:e2157.

14. Kjærbølling I, Vesth TC, Frisvad JC, Nybo JL, Theobald S, Kuo A, et al. Linking secondary metabolites to gene clusters through genome sequencing of six diverse *Aspergillus* species. Proc Natl Acad Sci. 2018;115:E753–61.

15. Quick J. Ultra-long read sequencing protocol for RAD004 v3 (protocols.io.mrxc57n) [Internet]. [cited 2019 Oct 25]. Available from: https://www.protocols.io/view/ultra-long-read-sequencing-protocol-for-rad004-mrxc57n

16. Wick R. rrwick/Porechop [Internet]. 2019 [cited 2019 Dec 3]. Available from: https://github.com/rrwick/Porechop

17. Marijon P, Chikhi R, Varré J-S. yacrd and fpa: upstream tools for long-read genome assembly. bioRxiv. 2019;674036.

18. Kiełbasa SM, Wan R, Sato K, Horton P, Frith MC. Adaptive seeds tame genomic sequence comparison. Genome Res. 2011;21:487–93.

19. Krzywinski M, Schein J, Birol İ, Connors J, Gascoyne R, Horsman D, et al. Circos: An information aesthetic for comparative genomics. Genome Res. 2009;19:1639–45.

20. Li H. Minimap2: pairwise alignment for nucleotide sequences. Bioinformatics. 2018;34:3094–100.

21. Li H, Handsaker B, Wysoker A, Fennell T, Ruan J, Homer N, et al. The Sequence Alignment/Map format and SAMtools. Bioinforma Oxf Engl. 2009;25:2078–9.

22. Thorvaldsdóttir H, Robinson JT, Mesirov JP. Integrative Genomics Viewer (IGV): high-performance genomics data visualization and exploration. Brief Bioinform. 2013;14:178–92.

23. Koren S, Walenz BP, Berlin K, Miller JR, Bergman NH, Phillippy AM. Canu: scalable and accurate long-read assembly via adaptive k-mer weighting and repeat separation. Genome Res. 2017;27:722–36.

24. Altschul SF, Gish W, Miller W, Myers EW, Lipman DJ. Basic local alignment search tool. J Mol Biol. 1990;215:403–10.

25. Katoh K, Standley DM. MAFFT multiple sequence alignment software version 7: improvements in performance and usability. Mol Biol Evol. 2013;30:772–80.

26. Gouy M, Guindon S, Gascuel O. SeaView Version 4: A Multiplatform Graphical User Interface for Sequence Alignment and Phylogenetic Tree Building. Mol Biol Evol. 2010;27:221–4.

27. Vaser R, Sović I, Nagarajan N, Šikić M. Fast and accurate de novo genome assembly from long uncorrected reads. Genome Res. 2017;27:737–46.

28. Kurtz S, Phillippy A, Delcher AL, Smoot M, Shumway M, Antonescu C, et al. Versatile and open software for comparing large genomes. Genome Biol. 2004;5:R12.

Figure 1

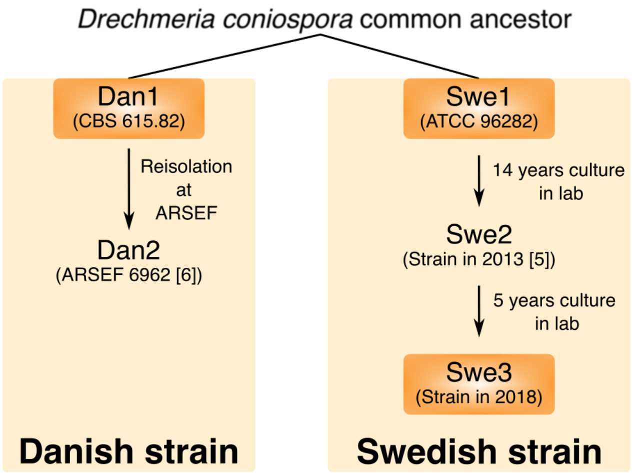
Drechmeria coniospora common ancestor
Dan1
(CBS 615.82)
Reisolation
at
ARSEF
Dan2
(ARSEF 6962 [6])
Danish strain
Swe1
(ATCC 96282)
14 years culture
in lab
Swe2
(Strain in 2013 [5])
5 years culture
in lab
Swe3
(Strain in 2018)
Swedish strain

Figure 2

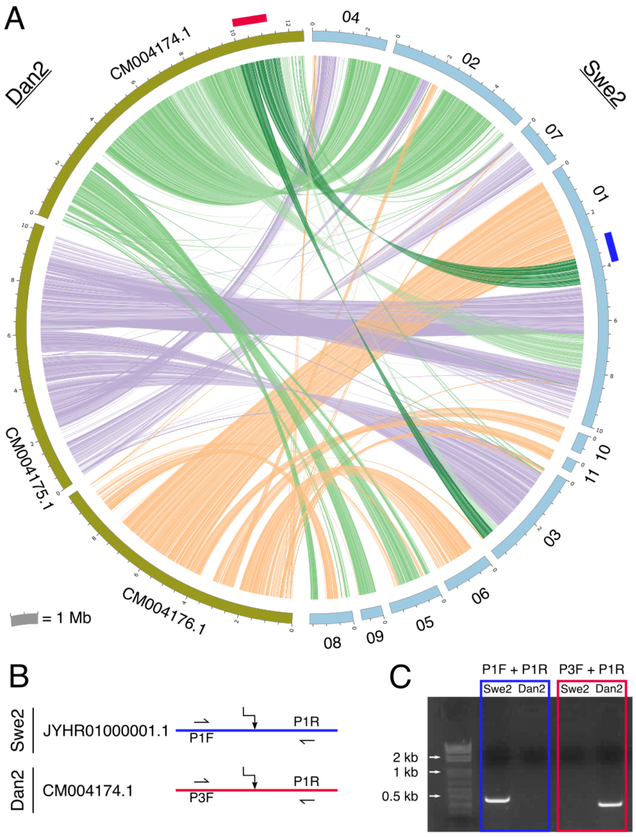
A
Dan2
CM004174.1
CM004175.1
CM004176.1
Swe2
04
02
07
01
10
11
03
06
05
09
08
= 1 Mb
B
Swe2
JYHR01000001.1
P1F
P1R
Dan2
CM004174.1
P3F
P1R
C
P1F + P1R
P3F + P1R
Swe2
Dan2
Swe2
Dan2
2 kb
1 kb
0.5 kb

Figure 3

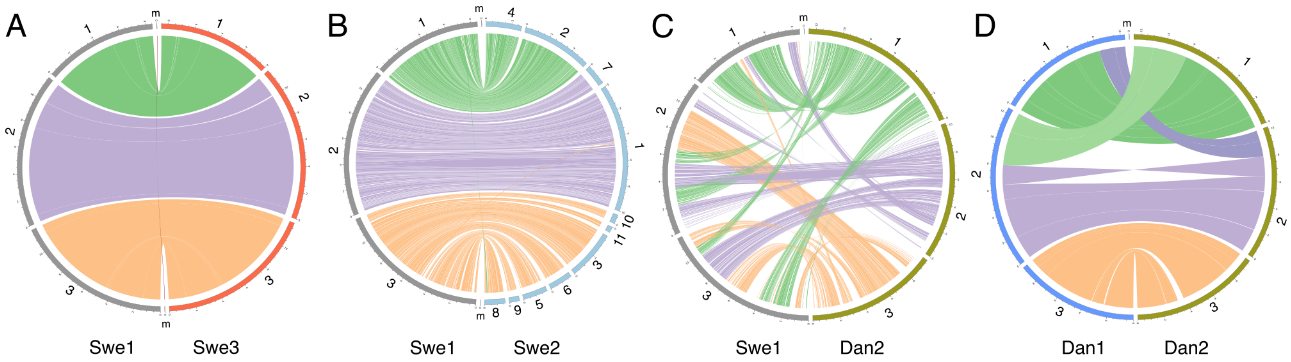

Figure 4

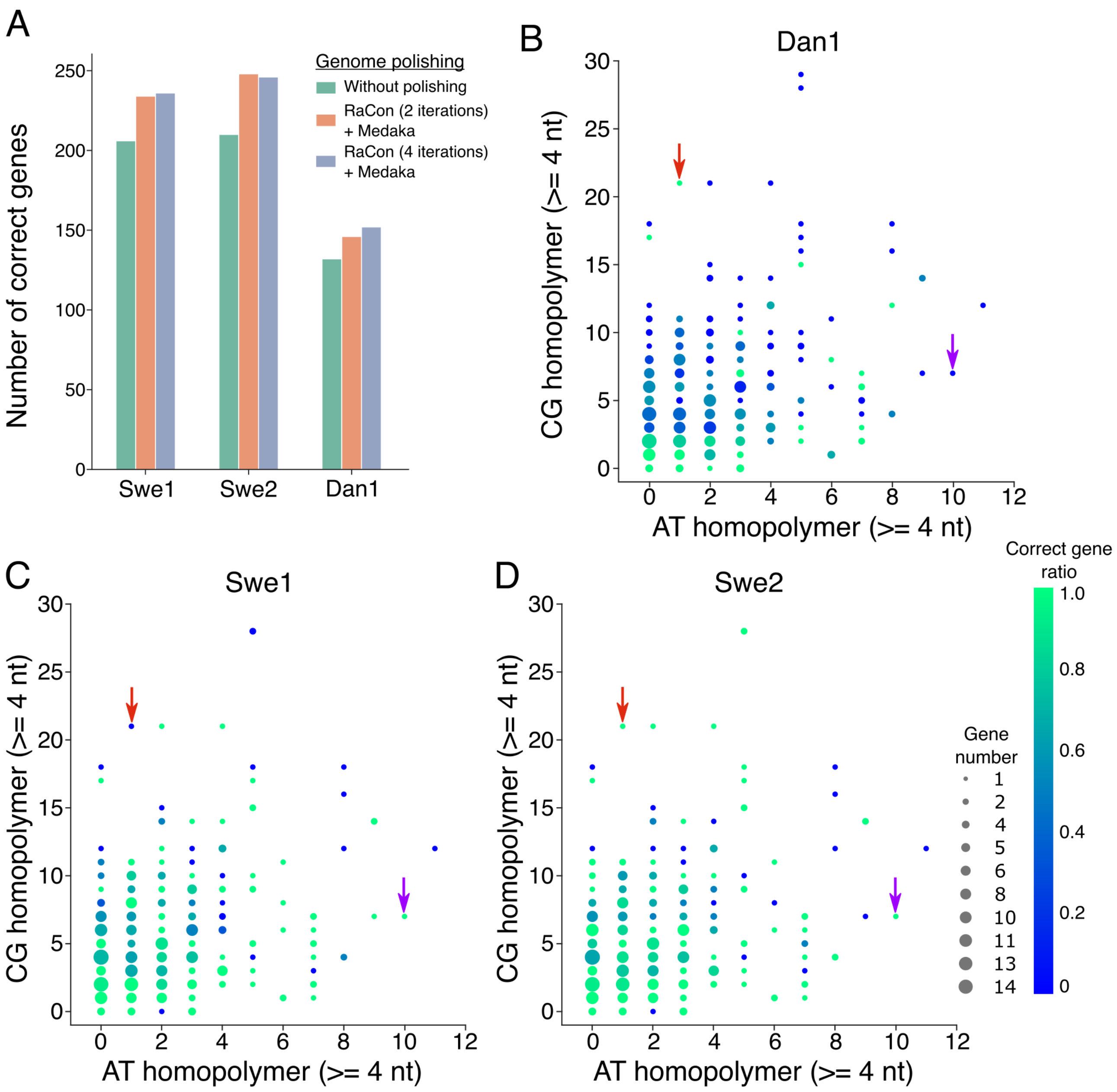
A
Genome polishing
Without polishing
RaCon (2 iterations) + Medaka
RaCon (4 iterations) + Medaka
Number of correct genes
Swe1
Swe2
Dan1
B
Dan1
C
Swe1
D
Swe2
CG homopolymer (>= 4 nt)
AT homopolymer (>= 4 nt)
Correct gene ratio
Gene number

Figure 5

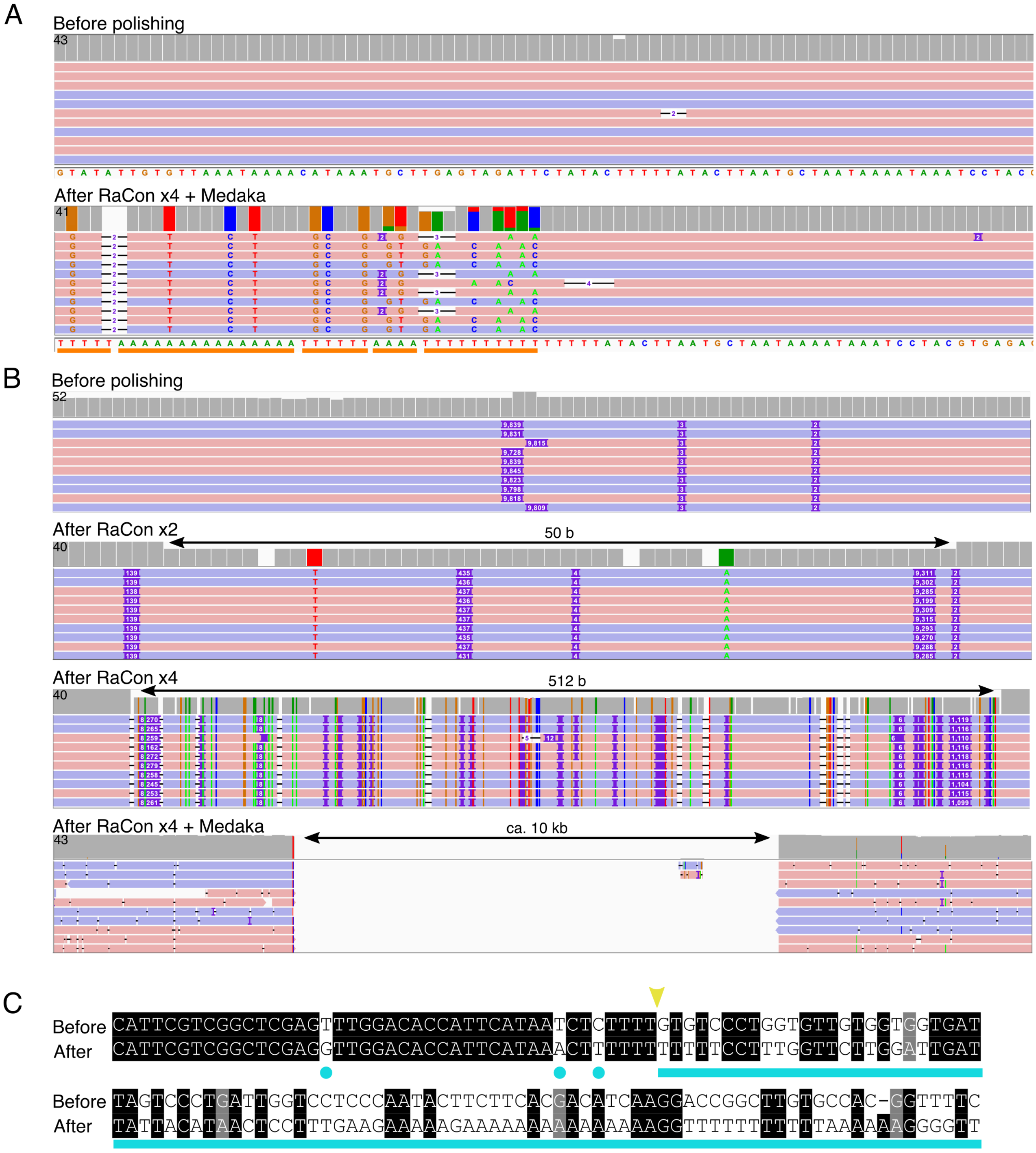
A
Before polishing
43
After RaCon x4 + Medaka
41
B
Before polishing
52
After RaCon x2
50 b
40
After RaCon x4
512 b
40
After RaCon x4 + Medaka
ca. 10 kb
43
C
Before CATTCGTCGGCTCGAGTTTGGACACCATTCATAATCTCTTTTGTGTCCCTGGTGTTGTGGTGGTGAT
After CATTCGTCGGCTCGAGGTTGGACACCATTCATAAACTTTTTTTTTTCCTTTGGTTCTTGGATTGAT
Before TAGTCCCTGATTGGTCCTCCCAATACTTCTTCACGACATCAAGGACCGGCTTGTGCCAC-GGTTTTC
After TATTACATAACTCCTTTGAAGAAAAAGAAAAAAAAAAAAAAAGGTTTTTTTTTTAAAAAAAGGGTT

Supplementary figure 1

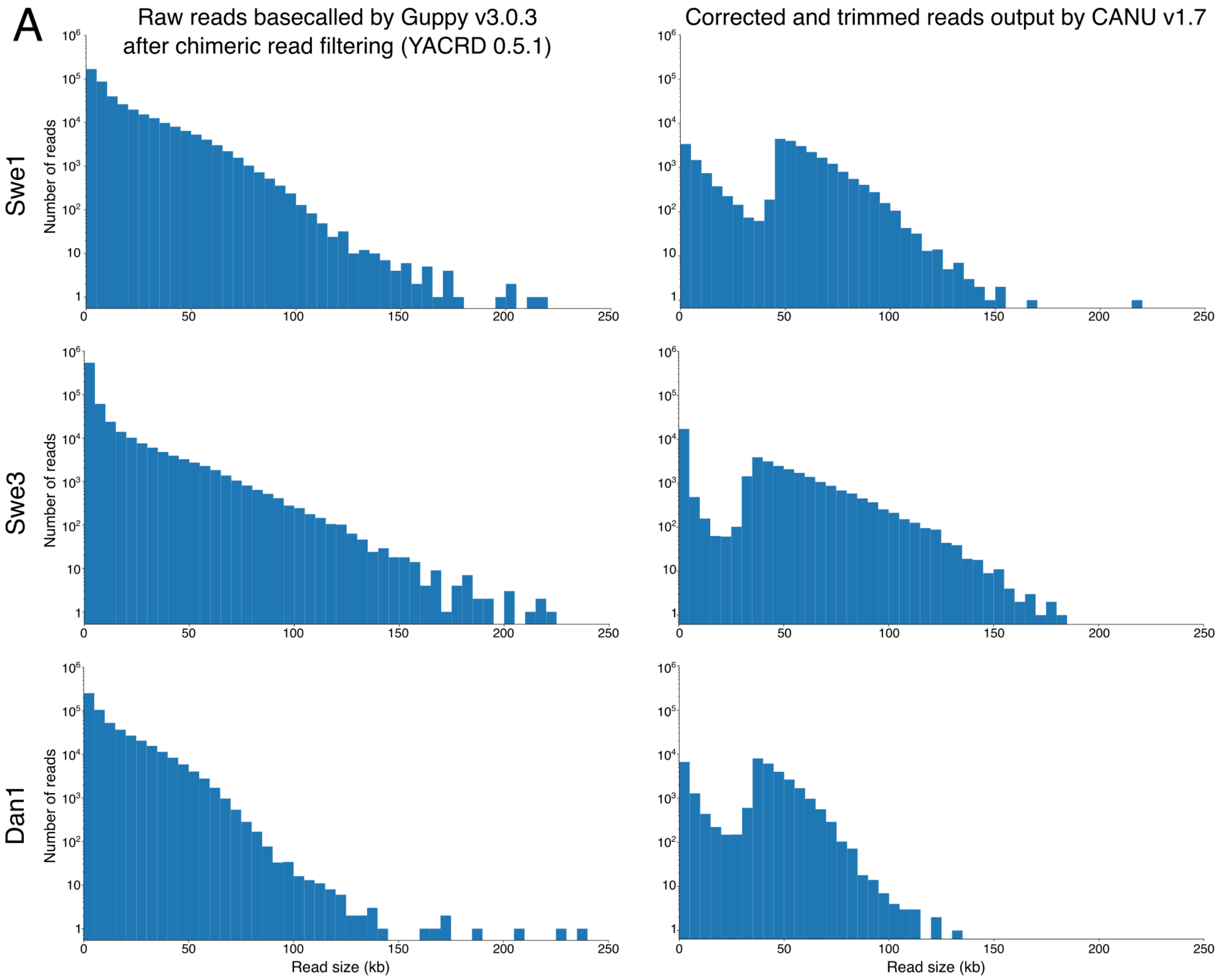

B

	Swe1		Swe3		Dan1	
	Canu assembly	after polishing*	Canu assembly	after polishing*	Canu assembly	after polishing*
Number of contigs	11	4	11	4	11	4
GC content (%)	55	55	55	55	55	55
N50 (b)	18 459 223	11 869 075	10 419 380	11 915 235	9 281 954	12 401 776
Total length (b)	32 676 850	32 121 045	32 440 698	32 271 010	32 353 052	32 120 031

*: RaCon x4 and Madaka on the manually curated assembly

Supplementary Figure 1. Distribution of the size of the reads and the assembly statistics.

A. Distributions in 5 kb bins of the size of the set of reads basecalled by Guppy v3.0.3, cleaned by Porechop v0.2.4, and after chimeric read filtering using YACRD v0.5.1 (lefthand panels), and of reads corrected and trimmed by Canu for the initial assemblies (righthand panels). B. General statistics for the Canu assemblies of Swe1, Swe3 and Dan1, and after manual curation and polishing.

Supplementary figure 2

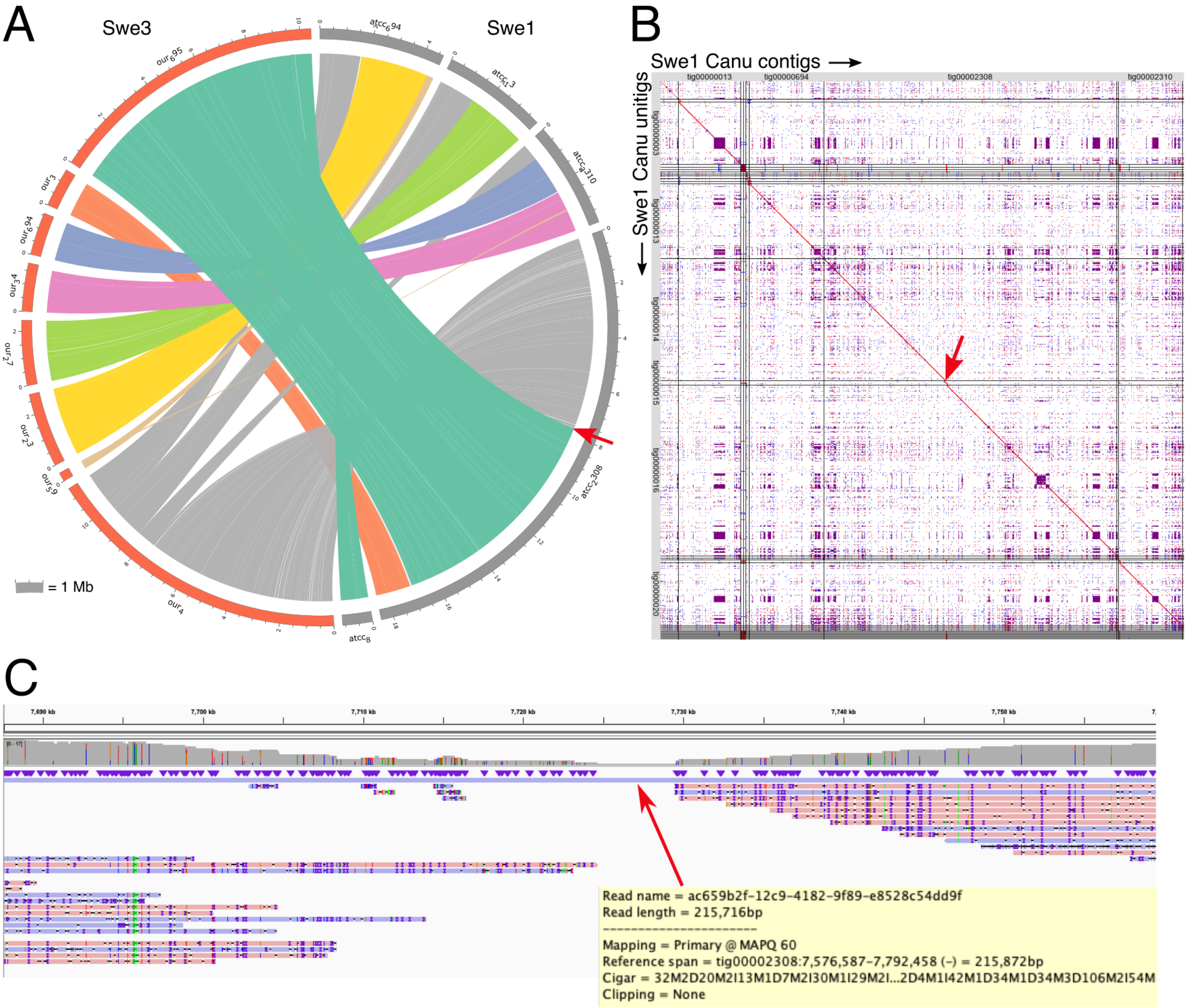

Supplementary Figure 2. Detection of a long chimeric read in the initial Swe1 assembly.

A. Circos plot representing regions >20 kb that are very similar between Swe3 and Swe1 Canu assemblies as determined by an all-against-all LAST analysis. The red arrow indicates a break in the synteny for the largest Swe1 contig. B. Dot-plot of an all-against-all comparison of the Swe1 contigs and unitigs produced by Canu. “Contig” means contiguous sequences present in the primary assembly, including both unique and repetitive elements. Unitigs are contigs split at alternate paths in the assembly graph. The red arrow indicates the discontinuity in the alignment between Swe1 contigs and unitigs. This occurs on the same contigs and at the same coordinates as in A. C. Mapping of the Swe1 reads, corrected and trimmed by Canu, on the Swe1 Canu assembly (detail of around 70 kb on contig tig00002308 flanking the synteny break). Moving into the central 5kb region, read support progressively drops from 35-40 to just one, corresponding to a very long read of about 215 kb, which was shown to be chimeric.

Supplementary figure 3

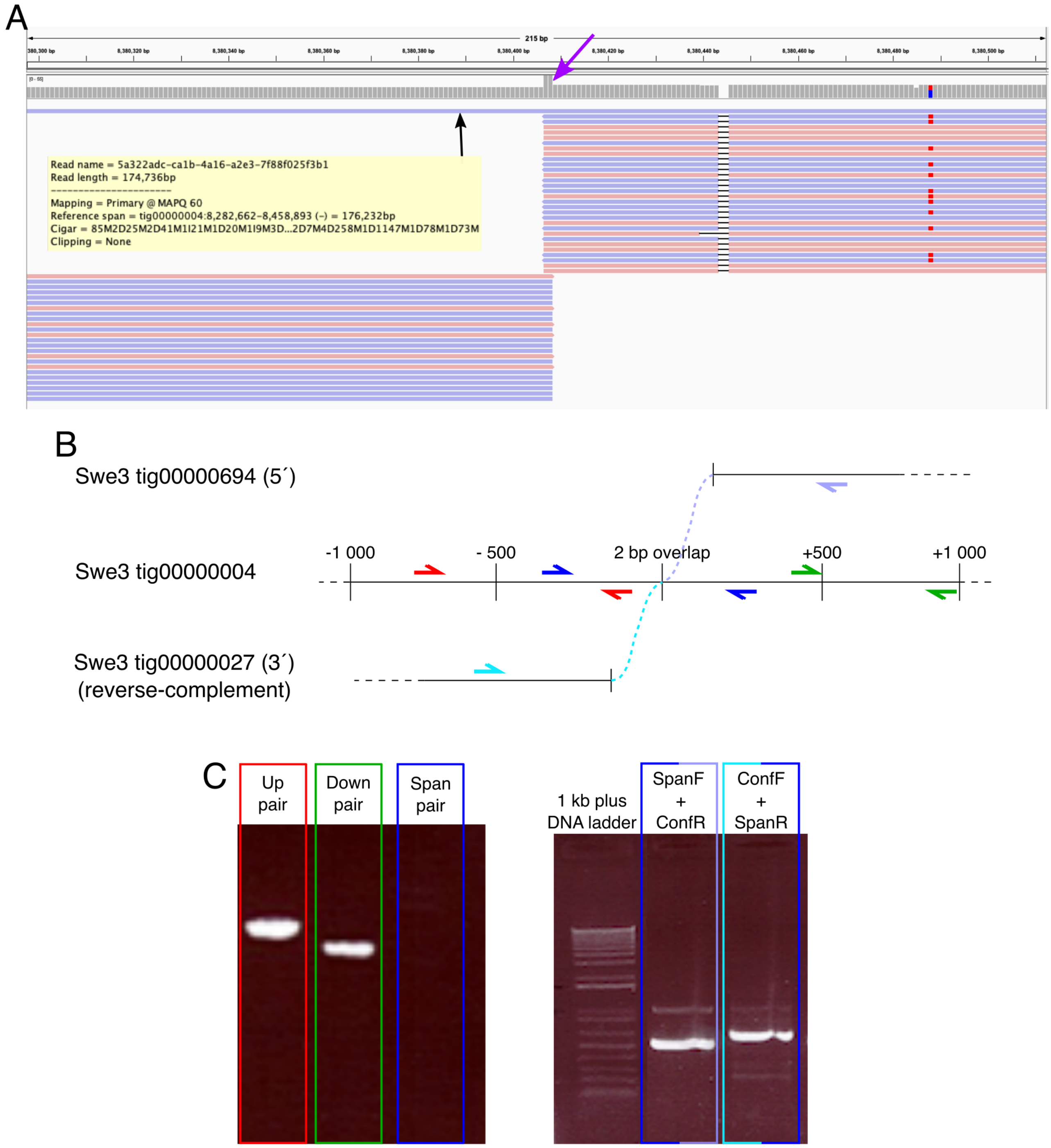

Supplementary Figure 3. Identification of a long chimeric read in the Swe3 initial assembly.

A. Mapping of the Swe3 reads, corrected and trimmed by Canu, on the Swe3 Canu assembly. The putative 175 kb chimeric read was identified by a break of synteny (not shown) and because of a sharp (ca. 2-fold) increase in coverage, spanning only 2 nucleotides, (purple arrow) on the contig tig0000004. B. Conceptual design of the PCR primers used to verify the assembly. Three pairs of primers were designed on the tig0000004: the Up pair (red), the Down pair (green) and the Span pair (blue). Two other primers were designed on the basis of the corrected assembly sequence (dotted coloured lines): SpanF in turquoise on the contig tig00000027 and SpanR in light purple on the contig tig00000695. C. PCR result of the different pairs used. Amplicons had the expected sizes.

Supplementary figure 4

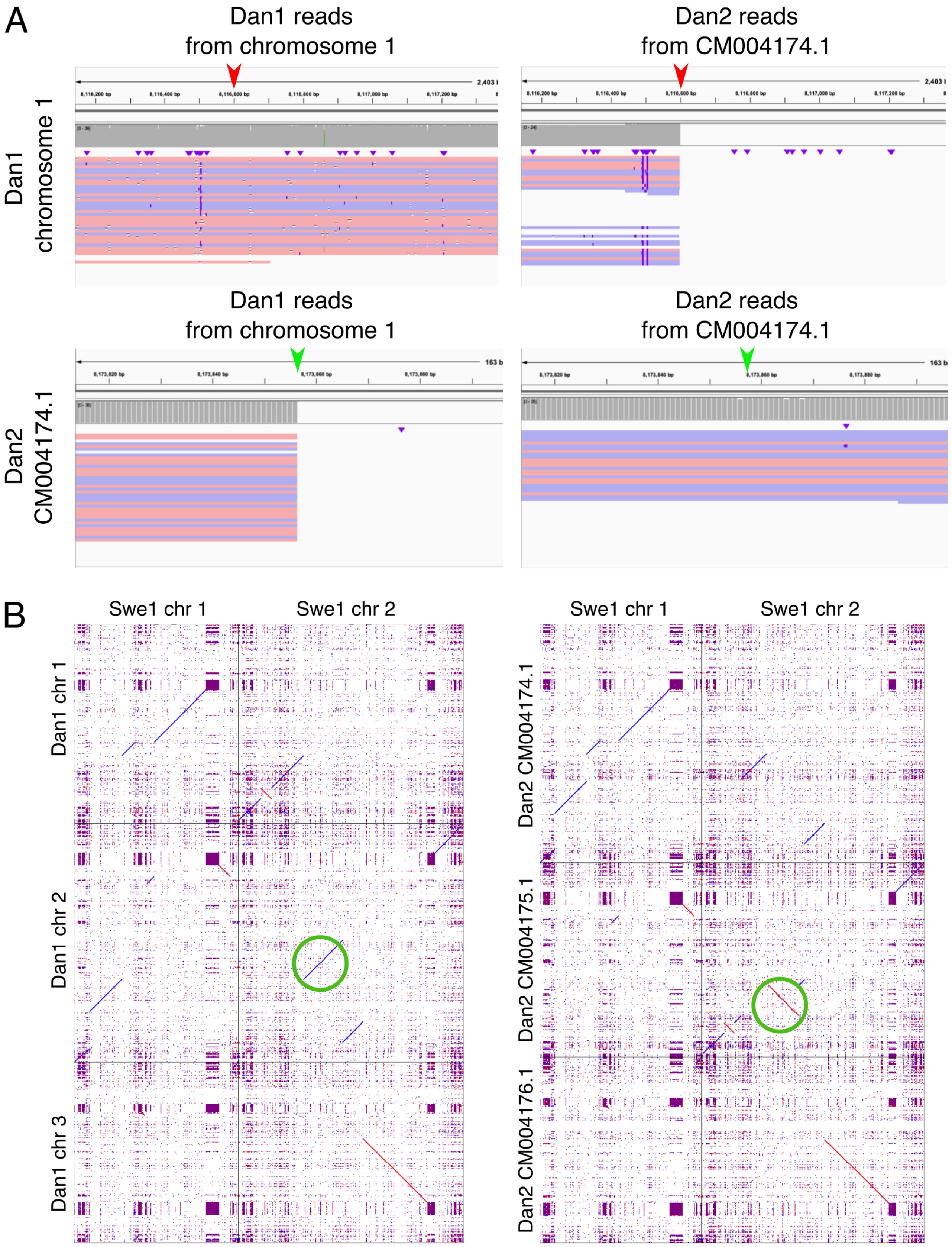

Supplementary Figure 4. Identification of intra- and inter-chromosomal rearrangements between Dan1 and Dan2.

A. Mapping of long reads from Dan1 (left panels) and Dan2 (right panels) on Dan1 chromosome 1 (top panels) and Dan2 chromosome 1 (CM004174.1; bottom panels). The arrowheads highlight points of discontinuity in the read coverage, consistent with chromosomal rearrangements between Dan1 and Dan2. B. Alignment of selected chromosomes of the final assemblies between Dan1 and Swe1 (left) and Dan2 and Swe1 (right). The unique difference in the orientation of part of Swe1 chromosome 2 between Dan1 and Dan2 is highlighted by the green circles.

Supplementary methods

GenBank query:

We queried the Assembly database on the NCBI website with:

(((("minion"[Sequencing Technology] OR "nanopore"[Sequencing Technology] OR "nanopore minion"[Sequencing Technology] OR "nanopore technologies"[Sequencing Technology] OR "nanopore technology"[Sequencing Technology] OR "nanpore"[Sequencing Technology] OR "ont"[Sequencing Technology] OR "ont minion"[Sequencing Technology] OR "oxford"[Sequencing Technology] OR "oxford nanopore"[Sequencing Technology] OR "oxford nanopore minion"[Sequencing Technology] OR "oxford nanopore technologies"[Sequencing Technology] OR "oxford nanopore technology"[Sequencing Technology]))) NOT illumina) NOT bgi

We refined the search with several filters on this query: Latest, Latest GenBank, Exclude derived from surveillance project, Exclude anomalous. Sequence records were checked to confirm that they had been generated using ONT long reads.

PCR

PCR was done to test the chimeric nature of a Canu contig due do a putative chimeric read in the Swe3 assembly, with primers UpF (AACTGTGTCTAACTAGCCCG), UpR (AGGGTCCTCATAAACTTGGC), DownF (TGTATCAGGTTCCCGAATGG), DownR (CTAGGCTGGGGAATCTTCTG), SpanF (CCATCAACTTCAGCTGCTC), SpanR (CTCCTCAATCTCCCTCTCGG), ConfF (ATCGGCGACTACCTGCAC), ConfR (CGTTCCATCGTTACCACAGC). PCR

reactions were run according to the GoTaq® G2 Flexi DNA polymerase instructions (Promega), with 50 ng of template DNA, 1 mM of each forward and reverse primers, in a final volume of 25 μL. The reaction started by initial denaturation at 95°C for 2 min, followed by 30 amplification cycles (95°C for 30 sec, 60°C for 30 sec and 72°C for 30 sec), and a final elongation for 5 min at 72°C.

Supplementary table 1

##Comments

#gene Gene invol\ ID refer to Swe2 protein ID

#index_hom simple index to help sorting homopolymer based on their order of appearance on Swe1 genome"

#letter Letter of the homopolymer

#Swe2 Homopolymer length in Swe2 (reference for Swe1 and Swe3)

#Swe1 Homopolymer length in Swe1

#Swe1_diff_ Do the length is different between this strain and its reference?

#Swe1_read Number of read present in the neighbor of this homopolymer - CANU set

#Swe1_read Number of read present in the strain supporting its homopolymer length - CANU set

#Swe1_gupp A remark on the support of this homopolymer in the full set of reads that was used to polish the genome - Guppy 3.0.3

#Swe1_gupp Ratio of reads supporting the alternate number of homopolymer

#Swe3 Homopolymer length in Swe3

#Swe3_diff_ Do the length is different between this strain and its reference?

#Swe3_read Number of read present in the neighbor of this homopolymer - CANU set

#Swe3_read Number of read present in the strain supporting its homopolymer length - CANU set

#Swe3_gupp A remark on the support of this homopolymer in the full set of reads that was used to polish the genome - Guppy 3.0.3

#Swe3_gupp Ratio of reads supporting the alternate number of homopolymer

#zhang Homopolymer length in Dan2 (Reference for Dan1)

#Dan1 Homopolymer length in Dan1

#Dan1_diff_ Do the length is different between this strain and its reference?

#Dan1_read_ Number of read present in the neighbor of this homopolymer - CANU set

#Dan1_read_ Number of read present in the strain supporting its homopolymer length - CANU set

#Dan1_gupp A remark on the support of this homopolymer in the full set of reads that was used to polish the genome - Guppy 3.0.3

#Dan1_gupp Ratio of reads supporting the alternate number of homopolymer

#

90% accuracy in read count

##Table

gene	index_homopol	letter	Swe2	Swe1	Swe1_diff_nbr	Swe1_read_tot	Swe1_read_support	Swe1_guppy_rq	Swe1_guppy_ratio	Swe3	Swe3_diff_nbr	Swe3_read_tot	Swe3_read_support	Swe3_guppy_rq	Swe3_guppy_ratio	Dan2	Dan1	Dan1_diff_nbr	Dan1_read_tot	Dan1_read_support	Dan1_guppy_rq	Dan1_guppy_ratio
ODA80475.1	1	c	5	5	-	36	2	supported	-	5	-	34	0	15/83 suppo	-	5	6	yes	28	0	5/135 suppo	0.04
ODA80475.1	2	t	4	4	-	35	0	supported	-	4	-	34	3	30/80 suppo	-	4	4	-	28	12	supported	-
ODA80475.1	3	t	NA	NA	NA	NA	NA	NA	NA	NA	NA	NA	NA	NA	NA	4	4	-	28	3	supported	-
ODA80475.1	4	c	4	4	-	37	23	supported	-	4	-	35	33	supported	-	4	5	yes	27	2	27/135 supp	0.20
ODA80475.1	5	g	7	8	yes	36	0	6/143 suppo	0.04	6	yes	35	2	21/82 suppo	0.26	7	7	-	28	0	supported	-
ODA80133.1	6	c	4	4	-	33	33	supported	-	4	-	41	41	supported	-	4	4	-	37	36	supported	-
ODA80133.1	7	c	4	4	-	33	26	supported	-	4	-	41	38	supported	-	4	4	-	36	18	supported	-
ODA80133.1	8	g	4	4	-	33	32	supported	-	4	-	42	40	supported	-	4	4	-	35	35	supported	-
ODA80133.1	9	c	7	6	yes	32	0	30/140 supp	0.21	6	yes	41	0	24/98 suppo	0.24	7	7	-	37	0	supported	-
ODA83792.1	10	c	4	4	-	33	33	supported	-	4	-	38	38	supported	-	4	4	-	28	28	supported	-
ODA83792.1	11	c	4	4	-	33	30	supported	-	4	-	38	36	supported	-	4	4	-	28	24	supported	-
ODA83792.1	12	c	4	4	-	33	32	supported	-	4	-	38	34	supported	-	4	4	-	28	28	supported	-
ODA83792.1	13	c	5	6	yes	33	0	11/124 supp	0.09	5	-	38	17	supported	-	5	6	yes	28	0	15/155 supp	0.10
ODA83792.1	14	c	4	4	-	33	33	supported	-	4	-	38	36	supported	-	4	4	-	28	27	supported	-
ODA83792.1	15	c	4	4	-	32	32	supported	-	4	-	38	36	supported	-	4	4	-	28	28	supported	-
ODA83792.1	16	c	5	5	-	32	0	60/125 supp	-	5	-	38	0	20/90 suppo	-	5	5	-	28	1	supported	-
ODA83792.1	17	c	5	5	-	32	12	50/120 supp	-	6	yes	38	0	8/90 support	0.09	5	5	-	28	11	supported	-
ODA83792.1	18	c	4	4	-	32	32	supported	-	4	-	38	37	supported	-	4	4	-	28	28	supported	-
ODA83792.1	19	c	4	4	-	32	31	supported	-	4	-	38	36	supported	-	4	4	-	28	28	supported	-
ODA83792.1	20	a	4	4	-	33	28	supported	-	4	-	37	35	supported	-	4	4	-	28	27	supported	-
ODA83792.1	21	a	4	4	-	33	28	supported	-	4	-	37	37	supported	-	4	4	-	28	21	supported	-
ODA83792.1	22	g	4	4	-	32	32	supported	-	4	-	38	37	supported	-	4	4	-	28	28	supported	-
ODA83792.1	23	t	4	4	-	32	32	supported	-	4	-	38	38	supported	-	4	4	-	28	28	supported	-
ODA83726.1	24	c	4	5	yes	25	0	18/120 supp	0.15	4	-	37	12	supported	-	4	5	yes	37	0	20/150 supp	0.13
ODA83726.1	25	c	4	4	-	25	23	supported	-	4	-	37	35	supported	-	4	4	-	37	36	supported	-
ODA83726.1	26	c	5	5	-	25	17	supported	-	5	-	37	27	supported	-	5	5	-	37	14	supported	-
ODA83726.1	27	c	5	6	yes	25	0	10/120 supp	0.1	5	-	37	12	supported	-	5	6	yes	37	0	18/150 supp	0.12
ODA83726.1	28	c	7	7	-	25	1	4/120 suppo	-	7	-	37	0	6/90 support	-	7	7	-	37	2	supported	-
ODA83726.1	29	c	5	5	-	25	24	supported	-	5	-	36	28	supported	-	5	5	-	37	26	supported	-
ODA83726.1	30	c	5	5	-	25	6	70/120 supp	-	5	-	36	13	supported	-	5	5	-	37	14	supported	-
ODA83726.1	31	g	4	4	-	25	24	supported	-	4	-	36	29	supported	-	4	4	-	37	20	supported	-
ODA84322.1	32	c	5	5	-	38	1	80/150 supp	-	5	-	41	0	supported	-	5	5	-	41	11	supported	-

ODA84322.1	33	g	4	4	-	38	29	supported	-	4	-	41	29	supported	-	4	4	-	41	21	supported	-
ODA84322.1	34	g	4	4	-	38	13	supported	-	4	-	40	7	supported	-	4	5	yes	41	1	45/180 supp	0.25
ODA84322.1	35	c	5	5	-	38	6	supported	-	5	-	42	3	supported	-	5	5	-	41	3	supported	-
ODA84322.1	36	c	5	5	-	38	6	supported	-	5	-	42	8	supported	-	5	5	-	41	1	supported	-
ODA84322.1	37	c	5	6	yes	38	0	13/150 supp	0.09	6	yes	42	3	11/110 supp	0.1	5	5	-	41	7	supported	-
ODA84322.1	38	g	4	4	-	37	37	supported	-	4	-	42	42	supported	-	4	5	yes	41	1	30/180 supp	0.17
ODA82449.1	39	c	5	5	-	39	4	70/140 supp	-	5	-	46	2	supported	-	5	5	-	26	8	supported	-
ODA82449.1	40	c	5	6	yes	39	1	8/140 suppo	0.06	6	yes	46	1	6/107 suppo	0.06	5	8	yes	26	0	2/160	0.01
ODA82449.1	41	c	5	5	-	39	16	supported	-	6	yes	46	2	6/107 suppo	0.06	5	5	-	26	8	supported	-
ODA82449.1	42	c	5	6	yes	39	0	5/138 suppo	0.04	6	yes	46	0	8/107 suppo	0.07	5	6	yes	26	0	6/160	0.04
ODA82449.1	43	a	5	5	-	38	21	supported	-	5	-	47	34	supported	-	5	5	-	26	6	supported	-
ODA82449.1	44	c	6	7	yes	38	0	3/140 suppo	0.02	7	yes	47	2	5/107 suppo	0.05	6	6	-	26	2	supported	-
ODA82449.1	45	c	5	5	-	38	25	supported	-	5	-	47	33	supported	-	5	5	-	26	15	supported	-
ODA81732.1	46	g	4	5	yes	19	0	20/140 supp	0.14	5	yes	40	0	17/110 supp	0.15	4	5	yes	39	0	40/180	0.22
ODA81732.1	47	c	7	7	-	20	1	5/135 suppo	-	7	-	40	2	10/107 supp	-	7	8	yes	40	1	10/180	0.06
ODA76966.1	48	c	5	5	-	40	1	60/150 supp	-	5	-	31	1	supported	-	5	6	yes	39	2	12/145	0.08
ODA76966.1	49	g	4	4	-	40	40	supported	-	4	-	31	30	supported	-	4	4	-	39	39	supported	-
ODA76966.1	50	c	4	4	-	40	32	supported	-	4	-	31	30	supported	-	4	4	-	39	29	supported	-
ODA76966.1	51	g	4	4	-	39	37	supported	-	4	-	31	30	supported	-	4	4	-	39	37	supported	-
ODA76966.1	52	c	4	4	-	39	31	supported	-	4	-	31	28	supported	-	4	4	-	39	29	supported	-
ODA76966.1	53	g	8	7	yes	38	0	11/150 supp	0.07	7	yes	31	1	3/90 support	0.03	8	7	yes	39	0	10/150	0.07
ODA76966.1	54	g	4	4	-	38	38	supported	-	4	-	31	31	supported	-	4	4	-	39	39	supported	-
ODA76653.1	55	c	4	4	-	39	37	supported	-	4	-	38	36	supported	-	4	4	-	40	39	supported	-
ODA76653.1	56	a	4	4	-	39	39	supported	-	4	-	38	38	supported	-	4	4	-	40	40	supported	-
ODA76653.1	57	g	4	4	-	39	39	supported	-	4	-	38	37	supported	-	4	4	-	40	40	supported	-
ODA76653.1	58	a	4	4	-	39	39	supported	-	4	-	38	38	supported	-	4	4	-	40	40	supported	-
ODA76653.1	59	g	4	4	-	39	37	supported	-	4	-	38	8	supported	-	4	4	-	40	38	supported	-
ODA78969.1	60	c	4	4	-	24	22	supported	-	4	-	29	28	supported	-	4	4	-	42	38	supported	-
ODA78969.1	61	c	4	4	-	23	23	supported	-	4	-	29	29	supported	-	4	4	-	42	42	supported	-
ODA78969.1	62	c	4	4	-	23	17	supported	-	4	-	29	20	supported	-	4	4	-	42	19	supported	-
ODA78969.1	63	c	4	4	-	23	22	supported	-	4	-	29	26	supported	-	4	5	yes	42	2	20/150	0.14
ODA78969.1	64	c	NA	NA	NA	NA	NA	NA	NA	NA	NA	NA	NA	NA	NA	6	6	-	42	2	supported	-
ODA78969.1	65	c	4	4	-	23	23	supported	-	4	-	29	29	supported	-	4	4	-	42	41	supported	-
ODA78969.1	66	c	7	6	yes	23	0	46/135 supp	0.34	6	yes	29	0	34/115 supp	0.28	7	6	yes	42	3	40/150	0.27
ODA78969.1	67	c	4	4	-	23	22	supported	-	4	-	29	29	supported	-	4	4	-	42	36	supported	-
