# Supplementary material for: Long-read only assembly of Drechmeria coniospora genomes reveals widespread chromosome plasticity and illustrates the limitations of current nanopore methods
Source: Gigascience. 2020 Sep 18;9(9):giaa099. doi: 10.1093/gigascience/giaa099 (PMC7500977; doi:10.1093/gigascience/giaa099)
Supplement: giaa099_Supplemental_Files [file giaa099_supplemental_files.zip › 03_supplementary_methods.pdf]

Supplementary methods

<u>GenBank query:</u>

We queried the Assembly database on the NCBI website with:

("minion"[Sequencing Technology] OR "nanopore"[Sequencing Technology] OR "nanopore minion"[Sequencing Technology] OR "nanopore technologies"[Sequencing Technology] OR "nanopore technology"[Sequencing Technology] OR "nanpore"[Sequencing Technology] OR "ont"[Sequencing Technology] OR "ont minion"[Sequencing Technology] OR "oxford"[Sequencing Technology] OR "oxford nanopore"[Sequencing Technology] OR "oxford nanopore minion"[Sequencing Technology] OR "oxford nanopore technologies"[Sequencing Technology] OR "oxford nanopore technology"[Sequencing Technology]) NOT (“illumina” OR “bgi” OR "pacbio" OR "pacbio rs" OR "pacbio rs ii" OR "pacbio rsii" OR "pacbio sequel" OR "pacbio smrt" OR "pacific biosciences" OR "sequel" OR "hybrid" OR "hybrid assembly") AND (latest[filter] OR "latest genbank"[filter]) AND (all[filter] NOT "derived from surveillance project"[filter]) AND (all[filter] NOT anomalous[filter])

The accuracy of this query depends on the assembly metadata in GenBank.

<u>K-mer based dot-plots:</u>

The dot-plots based on k-mers were computed with the script *repaver.r* (available at https://gitlab.com/gringer/bioinfscripts) The version used here was the commit 0854f3af76cb5bc5a7e0a2b4a032518f51e210fa, with the parameters *-k 11 -style dotplot*.

<u>Search for Dan1 / Dan2 transposable element at the breakpoints:</u>

A first analysis was conducted with the Dfam (https://dfam.org/home) to search for transposable elements (TE). The parameter *organism* was set to *other*. No TE was present in the neighbourhood of the different breakpoints. We also mined the two genomes for TE with transposonPSI (http://transposonpsi.sourceforge.net/) with default parameters. Here also, no TE was present within the 50kb surrounding each breakpoint.

PCR:

PCR was performed to test the putative chimeric nature of a Canu contig in the Swe3 assembly, with primers UpF (AACTGTGTCTAACTAGCCCG), UpR (AGGGTCCTCATAAACTTGGC), DownF (TGTATCAGGTTCCCGAATGG), DownR (CTAGGCTGGGGAATCTTCTG), SpanF (CCATCAACTTCAGCTGCTC), SpanR (CTCCTCAATCTCCCTCTCGG), ConfF (ATCGGCGACTACCTGCAC), ConfR (CGTTCCATCGTTACCACAGC). PCR reactions were run according to the GoTaq® G2 Flexi DNA polymerase instructions (Promega), with 50 ng of template DNA, 1 mM of each forward and reverse primers, in a final volume of 25 μL. The reaction started by initial denaturation at 95°C for 2 min, followed by 30 amplification cycles (95°C for 30 sec, 60°C for 30 sec and 72°C for 30 sec), and a final elongation for 5 min at 72°C.
