# Supplementary material for: Long-read only assembly of Drechmeria coniospora genomes reveals widespread chromosome plasticity and illustrates the limitations of current nanopore methods
Source: Gigascience. 2020 Sep 18;9(9):giaa099. doi: 10.1093/gigascience/giaa099 (PMC7500977; doi:10.1093/gigascience/giaa099)
Supplement: giaa099_Supplemental_Files [file giaa099_supplemental_files.zip › figure_suppl_legend.pdf]

Supplementary Figure 1. Distribution of the size of the reads and the assembly statistics.

Distributions in 5 kb bins of the size of the set of reads basecalled by Guppy v1.5.1(left-hand panels), and of reads corrected and trimmed by Canu for the initial assemblies (righthand panels).

Supplementary Figure 2. Detection of an error in the initial Swe1 assembly introduced by a long chimeric read.

A. Circos plot representing regions >20 kb that are very similar between Swe3 and Swe1 Canu assemblies as determined by an all-against-all LAST analysis. The red arrow indicates a break in the synteny for the largest Swe1 contig. B. Dot-plot of an all-against-all comparison of the Swe1 contigs and unitigs produced by Canu. Contigs are contiguous sequences present in the primary assembly, including both unique and repetitive elements. Unitigs are contigs split at alternate paths in the assembly graph. The red arrow indicates the discontinuity in the alignment between Swe1 contigs and unitigs. This occurs on the same contigs and at the same coordinates as in A. C. Mapping of the Swe1 reads, corrected and trimmed by Canu, on the Swe1 Canu assembly (detail of around 70 kb on contig tig00002308 flanking the synteny break). Moving into the central 5 kb region, read support progressively drops from 40 to just one, corresponding to a very long read of about 215 kb, which was shown to be chimeric.

Supplementary Figure 3. Detection of an error in the Swe3 initial assembly introduced by a long chimeric read.

A. Mapping of the Swe3 reads, corrected and trimmed by Canu, on the Swe3 Canu assembly. The putative 175 kb chimeric read was identified by a break of synteny (not shown) and because of a sharp (ca. 2-fold) increase in coverage, spanning only 2 nucleotides, (purple arrow) on the contig tig0000004. B. Conceptual design of the PCR primers used to verify the assembly. Three pairs of primers were designed on the tig0000004: the Up pair (red), the Down pair (green) and the Span pair (blue). Two other primers were designed on the basis of the corrected assembly sequence (dotted coloured lines): SpanF in turquoise on the contig tig00000027 and SpanR in light purple on the contig tig00000695. C. PCR result of the different pairs used. Amplicons had the expected sizes.

Supplementary Figure 4. Canu correction of non-chimeric and chimeric reads.

A. Scatter plots of the size of reads in the initial dataset (y-axis) and after Canu correction (x-axis) for non-chimeric (top) and chimeric (bottom) reads. Subsets of non-chimeric reads of a similar number (as indicated) and having the equivalent size distribution (before Canu correction, +/- 1% in length) as the chimeric read were used to allow a valid comparison. B. Percentage of read length reduction after Canu correction, in 5 kb bins. The bin size corresponds to the read size in the raw dataset. The red arrows highlight the rare chimeric reads that are longer after the correction step.

Supplementary Figure 5. Size distribution of chimeric reads.

Distributions in 5 kb bins of the size of the set of reads identified as chimeric by YACRD (see Methods), in the original dataset (left panels) and amongst reads basecalled by Guppy v3.0.3 (right panels).

Supplementary Figure 6. Analysis of the Swe1 chimeric reads.

For each chimeric read, the 500 bp of sequence on each side of the putative break point was mapped onto the final Swe1 genome. A, Distribution in 500 equal bins of the interval between the two parts of each chimeric read. The inserts highlight reads that map within 1 kb of each other (in 200 bins). B, Cumulative distribution of the interval between the two parts of each chimeric read (for those separated by > 1 Mb). The distance separating the two parts is broadly spread. C. Distribution of mapped sequences from each side of the putative breakpoint for each chimeric read that mapped to a single chromosome. The peak on chromosome 2 corresponds to the site where the nuclear genome matches the mitochondrial DNA, and that on chromosome 3 to a highly repeated region containing tandem copies of rDNA. In both cases, these therefore reflect erroneous attribution of chimerism. There are thus no true hot spots for chimeric read breakpoints on any chromosome.

Supplementary Figure 7. Identification of intra- and inter-chromosomal rearrangements between Dan1 and Dan2.

A. Mapping of long reads from Dan1 (left panels) and Dan2 (right panels) on Dan1 chromosome 1 (top panels) and Dan2 chromosome 1 (CM004174.1; bottom panels). The arrowheads highlight points of discontinuity in the read coverage, consistent with chromosomal rearrangements between Dan1 and Dan2. B. Alignment of selected chromosomes of the final assemblies between Dan1 and Swe1 (left) and Dan2 and Swe1 (right). The unique difference in the orientation of part of Swe1 chromosome 2 between Dan1 and Dan2 is highlighted by the green circles.

Supplementary Figure 8. Analysis of the pattern of repeated sequences in the neighbourhood of polishing error in the Swe3 genome.

A. Dot-plot based on k-mer identity (k = 11; See Supplementary Methods) of the neighbourhood of the *numts vs* itself, from the left to the right, before any polishing, after long-read polishing and after short-read polishing. The vertical dashed line at the centre of each plot indicates the start of the *numts*. B. A schematic representation of the large-scale genomic organization in the same region. The rectangles (red and dark red) represent *numts* (identified by BLASTN against the Swe3 mitochondrial genome) and the ribbons show the regions of sequence similarity (>99.5%) between them. For the Swe1 assembly, one dark-red rectangle is absent, while in the Swe2 assembly, the two dark-red rectangles are absent. C. An alignment of the area before any polishing (x-axis) and after short-read polishing (y-axis) reveals the re-inclusion of this missing sequence in the final assembly. The arrow highlights a 1.2 kb region that is duplicated on both sides of the sequence discontinuity (vertical dashed line). D. Dot-plots based on k-mers (k = 11) of the same region. The blue square reflects the presence of a region of low-complexity repeated sequences. The pattern at the top left hand of the plots reflect a region of low-complexity sequence that is not shared across the sequence discontinuity (vertical dashed line).
