# Supplementary material for: Long-read only assembly of Drechmeria coniospora genomes reveals widespread chromosome plasticity and illustrates the limitations of current nanopore methods
Source: Gigascience. 2020 Sep 18;9(9):giaa099. doi: 10.1093/gigascience/giaa099 (PMC7500977; doi:10.1093/gigascience/giaa099)
Supplement: giaa099_Response_to_Reviewer_Comments_Original_Submission [file giaa099_response_to_reviewer_comments_original_submission.pdf]

Author's Response To Reviewer Comments

Close

We thank the reviewers for their constructive criticisms. In the light of their comments, we have undertaken a substantial amount of work, resulting in 4 new supplementary figures (Figs S4, S5, S6, S8), and revision of 2 others (Fig. 4; Fig S1), as well as extensive changes to the text in the light of their remarks.

The principal criticism was the absence of accurate short-read data to produce high-quality genomes. We have addressed this issue by generating these datasets and have made this data as well as the polished whole genome sequences publicly available. The sequences have allowed us to validate fully our conclusions regarding the current state of ONT-only genome assemblies (see revised Figure 4, and new Supplementary Table 1). The polishing we have done has allowed us to address with more confidence the question of plasticity at the level of the DNA sequence, and we have included a general comparison of the polished genome sequences in the revised manuscript. A deeper exploration of the differences between the strains would involve generating a curated gene prediction for each genome and undertaking a detailed cataloguing of SNPs and indels. This is a project in itself, and although we are now embarking on this, we believe that it is outside the scope of the current manuscript.
With regards the overall comparisons, we now include a BUSCO analysis of the different assembly steps in the main text (new Table 1). As we point out, this kind of analysis should be treated with caution. In contrast to our careful examination of 305 mono-exonic genes, the BUSCO scores of the assemblies after long-read or short -read polishing were very similar since frame-shifts due to homopolymer length errors are not necessary picked up by BUSCO. We note in passing that the proportion of correct genes in the assemblies before polishing (Figure 4) is in reality even lower than originally presented. Due to an error in the data parsing script, we had used assemblies from a preliminary polishing test instead of assemblies without polishing. The results show that current polishing tools do a very good, but far from perfect job of error correction when using just nanopore reads. As one of the reviewers pointed out, ONT-only genomes merit being flagged in sequence databases.

Otherwise, Reviewer 1 was curious about the two long chimeric reads that caused mis-assemblies in Swe1 and Swe3, and more generally chimeric reads. We now show that chimeric reads are more likely to be trimmed by Canu, and to a greater extent, compared to non-chimeric reads (new Supplementary Figure 4). As requested by both reviewers, we also tried a different assembler, Flye. Although it is less sensitive to the presence of chimeric reads (new Supplementary Figure 6A), we detected other anomalies with this tool (new Supplementary Figure 6B-D). Our observations support the reviewers' suggestion that using more than one assembler can help determine correct genome structure.

With regards Reviewer 2's comments:

1. For the issue of short read polishing, see above. The different references have been included.
2. We now address the question of the quality of PacBio-only genomes in the main text.
3. We have expanded the section regarding the DNA extraction protocol and added information about read coverage (new Supplementary Table 1).
4. Concerning the loss of sequence accuracy after polishing, we addressed the question of whether the affected regions were repeat regions. Of the two examples of inconsistencies introduced by long-read polishing (Figure 5), the first was a region close to a nuclear insert of a fragment of the mitochondrial genome (also referred to as numts sequences), which in part is duplicated in tandem (new Supplementary Figure 8A-B). We conclude that the combination of this duplication plus the fact that reads from mitochondrial DNA can align in the region, led polishing tools to introduce erroneous sequence. The second case was about a chunk of ca. 10 kb omitted by Canu in the assembly of Swe3, but strongly supported by the reads. We had shown that polishing tools introduced a sequence with the correct length but with an erroneous composition. As presented in the new Supplementary Figure 8 C-D, there is indeed a small repeated sequence in the neighbourhood of the 10 kb chunk.
5. As requested we undertook a close examination of the rearrangement break points, but failed to detect any notable signature of transposable elements or repeated sequences in the neighbourhood (revised Supplementary Methods).

6. See comments about Flye, above

Close
