# Supplementary material for: Long-read only assembly of Drechmeria coniospora genomes reveals widespread chromosome plasticity and illustrates the limitations of current nanopore methods
Source: Gigascience. 2020 Sep 18;9(9):giaa099. doi: 10.1093/gigascience/giaa099 (PMC7500977; doi:10.1093/gigascience/giaa099)
Supplement: giaa099_Response_to_Reviewer_Comments_Revision_1 [file giaa099_response_to_reviewer_comments_revision_1.pdf]

Author's Response To Reviewer Comments

Close

As requested, we moved Figure S6 into the main text, to address the question of why BUSCO is not an appropriate tool added new data and a new figure (Figure 7), and present a detailed analysis of the nature of chimeric reads (new Supplementary Figure 6). Please see Cover Letter for full response.

Close
