# Supplementary material for: Long-read only assembly of Drechmeria coniospora genomes reveals widespread chromosome plasticity and illustrates the limitations of current nanopore methods
Source: Gigascience. 2020 Sep 18;9(9):giaa099. doi: 10.1093/gigascience/giaa099 (PMC7500977; doi:10.1093/gigascience/giaa099)
Supplement: giaa099_Reviewer_1_Report_Original_Submission — Jue Ruan -- 1/30/2020 Reviewed [file giaa099_reviewer_1_report_original_submission.pdf]

Reviewer Report

Title: Long-read only assembly of Drechmeria coniospora genomes reveals widespread chromosome plasticity and illustrates the limitations of current nanopore methods.

Version: Original Submission **Date:** 1/30/2020

Reviewer name: Jue Ruan

Reviewer Comments to Author:

The authors sequenced three strains of Drechmeria coniospora using ONT, and assembled them with CANU. By examining the differences among new assemblies and previous reported assemblies at both structure and base levels, they found 1) long reads helped to assemble complex genomic regions, 2) chimeric reads led mis-assemblies, 3) homopolymer errors in final polished assemblies made the poor gene prediction. They also suggested to be careful to use the reference genomes assembled with pure ONT reads. I agree with most of those findings and suggestions. However, I think the main criticisms raised by the authors on ONT were already known to community. Also, the authors should continue to polish their assembly using NGS reads to explore the genome plasticity.
Obviously, without polishing using NGS reads, pure ONT assemblies cannot be used for gene prediction. But I am curious about the two very long but chimeric reads which respectively caused mis-assemblies in Swe1 and Swe3. There might be many other chimeric reads but didn't bring mis-assembly in CANU. Or if uses other long reads assemblers, will chimeric reads bring mis-assemblies on Drechmeria coniospora?

Level of Interest

Please indicate how interesting you found the manuscript: Choose an item.

Quality of Written English

Please indicate the quality of language in the manuscript: Choose an item.

Declaration of Competing Interests

Please complete a declaration of competing interests, considering the following questions:

- Have you in the past five years received reimbursements, fees, funding, or salary from an organisation that may in any way gain or lose financially from the publication of this manuscript, either now or in the future?
- Do you hold any stocks or shares in an organisation that may in any way gain or lose financially from the publication of this manuscript, either now or in the future?
- Do you hold or are you currently applying for any patents relating to the content of the manuscript?

- Have you received reimbursements, fees, funding, or salary from an organization that holds or has applied for patents relating to the content of the manuscript?
- Do you have any other financial competing interests?
- Do you have any non-financial competing interests in relation to this paper?

If you can answer no to all of the above, write 'I declare that I have no competing interests' below. If your reply is yes to any, please give details below.

I declare that I have no competing interests

I agree to the open peer review policy of the journal. I understand that my name will be included on my report to the authors and, if the manuscript is accepted for publication, my named report including any attachments I upload will be posted on the website along with the authors' responses. I agree for my report to be made available under an Open Access Creative Commons CC-BY license (http://creativecommons.org/licenses/by/4.0/). I understand that any comments which I do not wish to be included in my named report can be included as confidential comments to the editors, which will not be published.

Choose an item.

To further support our reviewers, we have joined with Publons, where you can gain additional credit to further highlight your hard work (see: https://publons.com/journal/530/gigascience). On publication of this paper, your review will be automatically added to Publons, you can then choose whether or not to claim your Publons credit. I understand this statement.

Yes Choose an item.
