# Supplementary material for: Long-read only assembly of Drechmeria coniospora genomes reveals widespread chromosome plasticity and illustrates the limitations of current nanopore methods
Source: Gigascience. 2020 Sep 18;9(9):giaa099. doi: 10.1093/gigascience/giaa099 (PMC7500977; doi:10.1093/gigascience/giaa099)
Supplement: giaa099_Reviewer_1_Report_Revision_1 — Jue Ruan -- 4/27/2020 Reviewed [file giaa099_reviewer_1_report_revision_1.pdf]

Reviewer Report

Title: Long-read only assembly of Drechmeria coniospora genomes reveals widespread chromosome plasticity and illustrates the limitations of current nanopore methods.

Version: Revision 1 **Date:** 4/27/2020

Reviewer name: Jue Ruan

Reviewer Comments to Author:

Overall, the authors have improved the manuscript by performing genome polish using NGS data and compared the assemblies generated by different assemblers. However, the new manuscript still highlights the unsatisfying accuracy of ONT-only assembly, and the improvement of genome quality after polishing using ONT and NGS reads, which has been shown in many different works (Schmidt et. al.,2017, the Plant Cell; Belser et.al., 2018, Nature Plant; Michael et.al., 2018,Nature communications). In the light of previous works on ONT assembly, the novelty of this finding should be toned down.
One very interesting but often overlooked problem in assembly using long read is the mis-assembly derived from chimeric reads. It would be very novel and informative to deeply explore the assembly of the chimeric reads observed in this study, that is why I suggested the experiment using other assemblers to deeply explore this issue. The authors did provide Supplementary Figure 4 and 5 to show the basic stats. However, I don't think "The use of other genome assembly tools, and the comparison of assembly discrepancies is an additional method to produce high confidence genomes" in L204-L206 is enough to produce accurate genome structure. Because users will be confused when assemblers give inconsistent structures, they are expecting how to do with conflict in the absence of reference genome. Besides, the authors reported the assembly generated using flye but ignored the mis-assembly caused by chimeric reads in it.

Level of Interest

Please indicate how interesting you found the manuscript: Choose an item.

Quality of Written English

Please indicate the quality of language in the manuscript: Choose an item.

Declaration of Competing Interests

Please complete a declaration of competing interests, considering the following questions:

- Have you in the past five years received reimbursements, fees, funding, or salary from an organisation that may in any way gain or lose financially from the publication of this manuscript, either now or in the future?

- Do you hold any stocks or shares in an organisation that may in any way gain or lose financially from the publication of this manuscript, either now or in the future?
- Do you hold or are you currently applying for any patents relating to the content of the manuscript?
- Have you received reimbursements, fees, funding, or salary from an organization that holds or has applied for patents relating to the content of the manuscript?
- Do you have any other financial competing interests?
- Do you have any non-financial competing interests in relation to this paper?

If you can answer no to all of the above, write 'I declare that I have no competing interests' below. If your reply is yes to any, please give details below.

I declare that I have no competing interests

I agree to the open peer review policy of the journal. I understand that my name will be included on my report to the authors and, if the manuscript is accepted for publication, my named report including any attachments I upload will be posted on the website along with the authors' responses. I agree for my report to be made available under an Open Access Creative Commons CC-BY license (http://creativecommons.org/licenses/by/4.0/). I understand that any comments which I do not wish to be included in my named report can be included as confidential comments to the editors, which will not be published.

Choose an item.

To further support our reviewers, we have joined with Publons, where you can gain additional credit to further highlight your hard work (see: https://publons.com/journal/530/gigascience). On publication of this paper, your review will be automatically added to Publons, you can then choose whether or not to claim your Publons credit. I understand this statement.

Yes Choose an item.
