# Supplementary material for: Long-read only assembly of Drechmeria coniospora genomes reveals widespread chromosome plasticity and illustrates the limitations of current nanopore methods
Source: Gigascience. 2020 Sep 18;9(9):giaa099. doi: 10.1093/gigascience/giaa099 (PMC7500977; doi:10.1093/gigascience/giaa099)
Supplement: giaa099_Reviewer_2_Report_Original_Submission — Christian Ahrens -- 2/9/2020 Reviewed [file giaa099_reviewer_2_report_original_submission.pdf]

**Reviewer Report**

**Title: Long-read only assembly of Drechmeria coniospora genomes reveals widespread chromosome plasticity and illustrates the limitations of current nanopore methods.**

**Version: Original Submission** **Date:** 2/9/2020

**Reviewer name: Christian Ahrens**

**Reviewer Comments to Author:**

Reviewer comments for GIGA-D-19-00433
The manuscript by Courtine and co-workers entitled "Long-read only assembly of Drechmeria coniospora genomes reveals widespread chromosome plasticity and illustrates the limitations of current nanopore methods" addresses an important research topic of broad interest to the readership of GigaScience and beyond.
Large advances in assembly algorithms and NGS technologies, in particular third generation long read sequencing technologies from Pacific Biosciences and Oxford Nanopore Technologies, have led to the fact that the number of genome assemblies reported is growing exponentially (including thousands of prokaryotic genomes).
Given that many publications have advocated the use of a combination of long read sequences (known to have issues with homopolymer stretches) with highly accurate short read sequences, I was surprised that this was not done in this study. Nevertheless, one relevant take home message from the study (Discussion page 8) is that a certain number of genome assemblies that only rely on ONT data are being uploaded in public data repositories; ideally, such assemblies are flagged at the NCBI or users are at least made aware of the implications.
Comments
1. The major criticism with the paper and its take home message as presented is the question why the authors did not include higher quality short read data right from the beginning? For a superb, state of the art core facility like the EMBL, this would seem straight-forward.
For studies on complex, repeat-rich prokaryotes, the advantages of very long ONT reads to help to correctly place long, nearly-identical repeat sequences of 40 kb and above has been reported (Schmid et al, Nucleic Acids Research 2018; PMID: 30137508); this study and reports on eukaryotes e.g. from the Loman group (Jain et al Nat Biotechnology 2018Â¸ PMID: 29431738) have used short read (or PacBio) data to achieve very high quality genome assemblies of both prokaryotes and eukaryotes.
2. In my view, the message that 3rd generation long read sequencing from the ONT platform when used alone is not up to par to assemble complex eukaryotic genomes needs to be put a bit more into perspective. On page 8 (2nd paragraph) it is mentioned that ONT only assemblies are being added to public sequence repositories (52 eukaryotes, 99 bacteria). I agree that these should be flagged. However, other studies should be cited that have reported eukaryotic genome assemblies and that have relied on additional higher quality short read (e.g. the key study from Jain et al, Nature Biotechnology 2018 (PMID: 29431738)).
Would the authors conclude that assemblies based on PacBio data alone also do not merit inclusion into

databases? What are the factors that undermine the utility of long read ONT data when used alone the most, the existence of long homopolymer stretches, or the existence of long near-identical repeats that need to be treated carefully in order not to lower the assembly quality (see also point 4).
3. The ONT platform is very sensitive to the quality of the genomic DNA extracted. Therefore, the method section needs to specify in more detail which DNA extraction protocol has been used (currently, the reader is referred to REF #14). Similarly, I was missing in the result section the coverage of the genome assembly that was achieved by long ONT reads.
4. One key point is the fact that polishing can reduce the accuracy in certain regions in the assembly (see top paragraph on page 6). Are the regions where this was observed repeat regions? This aspect has been noted in a study on long-read based genome assemblies from metagenome samples (Somerville et al BMC Microbiology 2019 (PMID:31238873): "Moreover, great care needs to be taken when contigs are polished individually, since this can lead to the erroneous removal of true, natural sequence diversity due to cross mapping of reads in repeat regions (e.g., repeated sequences such as 16S rRNA operons, insertion sequences/transposases)".
It would be valuable to detail this result section a bit further and explore/mention this for the examples shown in Figure 5.
5. One analysis that would have perfectly fit for the message of this paper was the analysis of the borders of the regions of the sites of reorganization between the Dan1 and Dan2 genomes (see page 8 1st paragraph; this is mentioned as an outlook). Can't these be run through a repeat analysis or against families of common repeat elements?
6. Canu was the only assembly algorithm explored. I wonder whether other assemblers that work well with error-prone reads and near identical repeat sequences (e.g. Kolmogorv et al, Nat Biotechnol 2019; PMID:30936562) would be helpful in this respect.
Minor
Figure S1 -> the axis is not labeled (kb)
Page 5; remove empty spaces after citation [9].
Methods (page 10); adapt… then adaptor were then trimmed -> then adaptors were trimmed

**Level of Interest**

Please indicate how interesting you found the manuscript: Choose an item.

**Quality of Written English**

Please indicate the quality of language in the manuscript: Choose an item.

**Declaration of Competing Interests**

Please complete a declaration of competing interests, considering the following questions:

- Have you in the past five years received reimbursements, fees, funding, or salary from an organisation that may in any way gain or lose financially from the publication of this manuscript, either now or in the future?
- Do you hold any stocks or shares in an organisation that may in any way gain or lose financially from the publication of this manuscript, either now or in the future?
- Do you hold or are you currently applying for any patents relating to the content of the manuscript?
- Have you received reimbursements, fees, funding, or salary from an organization that holds or has applied for patents relating to the content of the manuscript?
- Do you have any other financial competing interests?
- Do you have any non-financial competing interests in relation to this paper?

If you can answer no to all of the above, write 'I declare that I have no competing interests' below. If your reply is yes to any, please give details below.

I declare no competing interests.

I agree to the open peer review policy of the journal. I understand that my name will be included on my report to the authors and, if the manuscript is accepted for publication, my named report including any attachments I upload will be posted on the website along with the authors' responses. I agree for my report to be made available under an Open Access Creative Commons CC-BY license (http://creativecommons.org/licenses/by/4.0/). I understand that any comments which I do not wish to be included in my named report can be included as confidential comments to the editors, which will not be published.

Choose an item.

To further support our reviewers, we have joined with Publons, where you can gain additional credit to further highlight your hard work (see: https://publons.com/journal/530/gigascience). On publication of this paper, your review will be automatically added to Publons, you can then choose whether or not to claim your Publons credit. I understand this statement.

Yes Choose an item.
