# Supplementary material for: Long-read only assembly of Drechmeria coniospora genomes reveals widespread chromosome plasticity and illustrates the limitations of current nanopore methods
Source: Gigascience. 2020 Sep 18;9(9):giaa099. doi: 10.1093/gigascience/giaa099 (PMC7500977; doi:10.1093/gigascience/giaa099)
Supplement: giaa099_Reviewer_2_Report_Revision_1 — Christian Ahrens -- 5/8/2020 Reviewed [file giaa099_reviewer_2_report_revision_1.pdf]

Reviewer Report

Title: Long-read only assembly of Drechmeria coniospora genomes reveals widespread chromosome plasticity and illustrates the limitations of current nanopore methods.

Version: Revision 1 **Date:** 5/8/2020

Reviewer name: Christian Ahrens

Reviewer Comments to Author:

Reviewer comments for GIGA-D-19-00433R1
The authors have done a very good job at addressing the various reviewer comments in a revised manuscript. More specifically, they have added a substantial amount of new analyses that now more thoroughly support the claims made (e.g., showing in the new Table 1 how different polishing steps affect the quality of a set of USCOs, including information on fragmented and missing genes and mentioning that these olishing steps have to be treated with caution in repeat regions; exploring other genome assemblers (here Flye) ans using some manual curation efforts to create a final assembly of higher quality; further exploring mis-assembled regions for the presence of certain classes of repeats and clarifying the origin of a long chimeric read that lead to a certain assembly artefact) and that are in line with observations made by other groups in the field.
Indeed, one of the key messages is that caution has to be exercised when dealing with ONT long-red only assemblies (i.e. without any polishing using more accurate short read data) in genome sequence repositories.
I support publication of the revised manuscript.

Level of Interest

Please indicate how interesting you found the manuscript: Choose an item.

Quality of Written English

Please indicate the quality of language in the manuscript: Choose an item.

Declaration of Competing Interests

Please complete a declaration of competing interests, considering the following questions:

- Have you in the past five years received reimbursements, fees, funding, or salary from an organisation that may in any way gain or lose financially from the publication of this manuscript, either now or in the future?
- Do you hold any stocks or shares in an organisation that may in any way gain or lose financially from the publication of this manuscript, either now or in the future?

- Do you hold or are you currently applying for any patents relating to the content of the manuscript?
- Have you received reimbursements, fees, funding, or salary from an organization that holds or has applied for patents relating to the content of the manuscript?
- Do you have any other financial competing interests?
- Do you have any non-financial competing interests in relation to this paper?

If you can answer no to all of the above, write 'I declare that I have no competing interests' below. If your reply is yes to any, please give details below.

I declare that I have no competing interests

I agree to the open peer review policy of the journal. I understand that my name will be included on my report to the authors and, if the manuscript is accepted for publication, my named report including any attachments I upload will be posted on the website along with the authors' responses. I agree for my report to be made available under an Open Access Creative Commons CC-BY license (http://creativecommons.org/licenses/by/4.0/). I understand that any comments which I do not wish to be included in my named report can be included as confidential comments to the editors, which will not be published.

Choose an item.

To further support our reviewers, we have joined with Publons, where you can gain additional credit to further highlight your hard work (see: https://publons.com/journal/530/gigascience). On publication of this paper, your review will be automatically added to Publons, you can then choose whether or not to claim your Publons credit. I understand this statement.

Yes Choose an item.
